# At the intersection of mind and climate change: integrating inner dimensions of climate change into policymaking and practice

**DOI:** 10.1007/s10584-022-03398-9

**Published:** 2022-07-15

**Authors:** Christine Wamsler, Jamie Bristow

**Affiliations:** 1 Lund University Centre for Sustainability Studies (LUCSUS), Lund, Sweden; 2 The Mindfulness Initiative, London, UK

**Keywords:** Sustainability, Policy integration, Relationality, Inner transformation, Inner transition, Climate change mitigation, Climate change adaptation, Climate anxiety, Paradigms

## Abstract

Dominant policy approaches have failed to generate action at anywhere near the rate, scale or depth needed to avert climate change and environmental disaster. In particular, they fail to address the need for a fundamental cultural transformation, which involves a collective shift in mindsets (values, beliefs, worldviews and associated inner human capacities). Whilst scholars and practitioners are increasingly calling for more integrative approaches, knowledge on how the link between our mind and the climate crisis can be best addressed in policy responses is still scarce. Our study addresses this gap. Based on a survey and in-depth interviews with high-level policymakers worldwide, we explore how they perceive the intersection of mind and climate change, how it is reflected in current policymaking and how it could be better considered to support transformation. Our findings show, on the one hand, that the mind is perceived as a victim of increasing climate impacts. On the other hand, it is considered a key driver of the crisis, and a barrier to action, to the detriment of both personal and planetary wellbeing. The resultant vicious cycle of mind and climate change is, however, not reflected in mainstream policymaking, which fails to generate more sustainable pathways. At the same time, there are important lessons from other fields (e.g. education, health, the workplace, policy mainstreaming) that provide insights into how to integrate aspects of mind into climate policies. Our results show that systematic integration into policymaking is a key for improving both climate resilience and climate responsiveness across individual, collective, organisational and system levels and indicate the inner human potential and capacities that support related change. We conclude with some policy recommendations and further research that is needed to move from a vicious to a virtuous cycle of mind and climate change that supports personal and planetary wellbeing.

**Supplementary Information:**

The online version contains supplementary material available at 10.1007/s10584-022-03398-9.

## Introduction

There is no shortage of rational arguments that support the need for urgent action to avert catastrophic climate change impacts. At the same time, dominant policy approaches have failed to generate action at anywhere near the rate, scale or depth needed (IPCC 2022a,b). This is despite 30 years of climate negotiations under the United Nations Framework Convention on Climate Change (UNFCCC) and associated policies and actions at local, national and sub-national levels (UNFCCC 2020a,b). Current approaches have focused on the external world of socio-economic structures, governance dynamics, economic incentives and technology (IPCC 2022a,b; Leichenko and O’Brien 2020) and fail to address the need for a more fundamental cultural transformation of society towards sustainability (Adger et al. 2013; Grušovnik 2012; Nielsen et al. 2021; O’Brien 2018; Rimanoczy 2014; Waddock 2015).

Scholars, policymakers and practitioners are thus increasingly calling for more integrative approaches that link inner and outer dimensions of climate change and address the minds or mindsets out of which cultures and systems arise (Conceição 2020; Figueres and Rivett-Carnac 2020; Göpel 2016; IPCC 2022a,b; Ives et al. 2020; Legrand et al. 2022; Parodi and Tamm 2018; Woiwode et al. 2021). Our minds/mindsets are the internal lens through which people see and navigate life. They include individual and collective values, beliefs, worldviews and associated inner (cognitive, emotional and relational) qualities/capacities (Wamsler et al. 2020, 2021).^1^ In accordance with systems theory, they are potential “deep” leverage points for transformation (Fischer and Riechers 2019; Ives et al. 2020; Meadows 1999; Woiwode et al. 2021). Whilst external interventions such as carbon taxes rest on “shallow” leverage points, addressing our minds is said to represent a “deep” leverage point with profoundly greater potential impact (ibid). At the same time, there is little comprehensive empirical and qualitative research on how our minds link to the climate crisis and policy across individual, collective and system levels; how they can become a leverage point for change; and how related considerations can be best addressed in our policy responses (Carter 2011; Grasso and Tàbara 2019; Ives et al. 2020; Köhler et al. 2019; Wamsler et al. 2021). Our study addresses this gap.

Despite important advances in fields such as environmental psychology, behavioural economics, sustainability science and education (e.g. American Psychological Association 2010; Bamberg and Möser 2007; Brundiers et al. 2021; Clayton 2019; Clayton and Manning 2018; Doherty and Clayton 2011; Hedlund-de Witt et al. 2014; Klöckner 2013), existing knowledge is still fragmented, and questions remain as to how different aspects of mind relate to climate policy and sustainability outcomes *across* individual, collective and system levels and vice versa (Parodi and Tamm  2018; Wamsler et al. 2021; Woiwode et al. 2021)*.* This is also reflected in calls for more integrative policy approaches and better-linking inner and outer transformation for sustainability, including in this year’s IPCC assessment reports (IPCC 2022a,b).

Against this background, the aim of the present study is to explore (i) policymakers’ understanding of the intersection of mind and climate change, (ii) how it is reflected in policymaking approaches and (iii) how it could be better considered in sustainability and climate work. Policymakers’ perspectives are the focus of this study as they are influential in initiating and propelling changes needed to address climate change. Based on a thorough systematisation of current thinking worldwide, our results show how our individual and collective minds are perceived as a victim and key driver for today’s climate crisis and condition the inner qualities/capacities needed to address it, resulting in a vicious cycle of deteriorating personal and planetary wellbeing. At the same time, they offer new evidence on how and why this understanding is not reflected in mainstream policymaking and how related constraints could be overcome. We discuss implications and conclude with some policy recommendations and further research needs.

## Methodology

This article presents the results of a research project conducted during 2020–2021, which involved in-depth interviews and consultations with politicians, political advisors and other policymakers who were working at the intersection of mind, climate and policymaking. The consultation process involved a survey and discussions of preliminary results and policy recommendations. Relevant networks, document reviews and snowball sampling were used to identify the survey participants.^2^ All respondents were asked to indicate any other relevant experts in the field, who were subsequently invited to participate in the study. This process resulted in 76 responses.

Interviews were held with information-rich, high-level policymakers; here, the aim was to gain in-depth insights regarding the focus of the study. Twenty-six interviews were held with people who were selected based on information given in the survey and document reviews. Inclusion criteria were an in-depth knowledge of climate policymaking approaches, mechanisms and structures and/or related aspects of mind (e.g. engagement in wellbeing and human development work). They included former high-level UN officials, UN policy advisors, Sustainable Development or Climate Policy Coordinators and Advisors within the European Commission (EC) or national states, parliamentarians and other politicians at national and transnational levels, international climate policy negotiators as well as Heads of Unit and advisors to EC directors or other public bodies on matters concerning human resources and wellbeing. See Suppl. Material A–D for the interview and survey questions and an overview of respondents.

In accordance with the three aims of the study, the survey and interviews addressed the following areas:

1. *The intersection of mind and climate change*: Based on their work and experience, how do respondents perceive the relationship between people’s minds and the climate crisis? How do they understand this relationship? What interactions come to mind?
2. *Links to climate policy/policymaking*: How is the identified intersection of mind and climate change addressed in current policymaking? How do respondents consider the intersection in their own work? What are their experiences and lessons learned at personal, collective, organisational and system levels?
3. *Future visions/pathways–addressing the gaps in current policymaking*: What are the drivers and barriers to advancing current approaches? What is their vision for improving current policymaking? What concrete measures and approaches can be taken?

In accordance with the research aims, the study did not aim to sample mainstream thinking nor to provide a representative picture of the wider policymaking sphere. Instead, the goal was to explore patterns of thinking about potential interactions between mind, climate change and policy in a selected, non-representative sample of experts from different fields and sectors. Interview data was gathered from the most information-rich sources available, in order to gain a picture that was as complete as possible. The consultative survey was used to subsequently calibrate and saturate the preliminary themes.

Thematic analysis was used to assess the survey data and interview transcripts (Braun and Clarke 2006; Nowell et al. 2017). It involved the following steps: (1) familiarisation with the data, (2) generating initial ideas and themes through open coding, (3) interpreting and systematically categorising the content into themes and associated patterns, (4) reviewing and (5) further defining through axial and selective coding. In addition, transformational capacities and climate mainstreaming frameworks were applied to cluster capacities, along with associated measures and approaches (IDG Initiative 2021; Wamsler 2015; Wamsler et al. 2021).^3^ The illustrative verbatim that are included in the following sections and in Suppl. Material E were anonymised to protect the privacy of participants.

Finally, as part of the consultation process, our preliminary results and policy recommendations were sent to and discussed with nine policymakers (four of whom had not been involved in the previous data collection). The nine policymakers were selected because of their extensive experience and insights gained from working with the UN, the EC and national governments. They acted as critical reviewers to validate our findings, refine their relevance for policymaking and reduce associated researcher bias. At the same time, we acknowledge the limitations accompanying our research approach that focuses on key informants working at the intersection of mind, climate and policymaking, thus not covering the full spectrum of opinions on the research topic.

## Results

### The intersection of mind and climate change

The results reveal that policymakers are beginning to consider the complex, intertwined nature of mind and climate change and how it translates into current policymaking. Respondents tended to identify climate change as a source of significant impacts on mental wellbeing (Sect. 3.1.1). At the same time, the mind itself is increasingly understood as a root cause of climate change (Sect. 3.1.2) and a barrier for action-taking (Sect. 3.1.3), resulting in a vicious cycle that leads to individual, societal and planetary deterioration (Sect. 3.1.4).

#### The mind—a victim of climate change

As described in more detail below, our findings demonstrate that climate change impacts our minds in three key ways. First, uncertainty and (potential for) catastrophic outcomes can affect mental health and wellbeing. Second, different ways of (non-)engaging with the issue can lead to feelings that reinforce such negative effects (e.g. denial, burnout). Third, mental impacts of climate change relate to its underlying drivers that further deteriorate wellbeing.

When asked about the intersection of mind and climate change, climate anxiety was generally the first issue that policymakers mentioned. Related terms and expressions included “eco-anxiety”, “climate grief”, “climate fear” and associated “overwhelm”. Most participants observed that there has been a considerable increase in such factors in society, particularly among younger generations, and suggested that it was a growing policy concern. They noted that “the situation that we’re in is creating a kind of drag on the overall mental health of a whole generation”. It was generally considered “a source of real concern” since “the mental stress associated with [climate change] is huge”, and “we’re only beginning to touch the surface of it”.

Many respondents perceived that the most devastating climate change impacts relate to mental health and stress. This was related to a constant feeling of uncertainty and unpredictability, fears about personal safety, traumatic experiences and losing a sense of identity, meaning and hope with long-term societal implications. These implications can include increasing drug abuse, interpersonal aggression, violence, crime, polarisation and extremism.

Many respondents also mentioned negative impacts resulting from the way people engage with climate change. Many feel that “it’s too big (…). They feel powerless (…) are losing sleep”. This can, in turn, lead to denial or guilt about not doing enough, even among those who engage until they burn out. In fact, feelings of stress, frustration, anxiety and ultimately burnout are also considered to be high in the respondents’ professional context, impacting and degrading their work in different ways. “The people that work on it day to day (…) do worry about it and carry that burden around with them (…). It’s one of those things that sits in the background and gnaws away”.

Finally, several respondents brought up the more indirect mental health impacts of the underlying social paradigms that drive climate change, which are described in detail in Sect. 3.1.2. They stated that “depression or anxiety is just to do with the way we live and how it fails to meet our mental health needs”. “Clearly, one needs a certain level of consumption (…), but beyond a certain level it leads to an undermining of wellbeing”.

#### The mind—a root cause of climate change

A less common, but increasingly prominent perspective is that the mind is described as being a key driver, or root cause, of the climate crisis. Three key arguments emerged, notably that climate change is an outward manifestation of exploitative mindsets. These mindsets are in turn rooted in a disconnect with ourselves (emotions, bodies), others and nature, and they shape, and are shaped by, the dominant social paradigms (economic growth and associated consumerism, materialism, competition and individualism), indicating the intertwined nature of our individual and collective mindsets. Respondents pointed out that “it’s the human mind that is at the heart of the climate crisis, even though it’s often not mentioned or spoken about”. However, related awareness is relatively low among policymakers and society as a whole. This is partly a result of the fact that “all of our institutions and networks, and the way we think about these issues is predicated on this rigid, huge wall between inner and outer”. Consequently, one respondent observed, “one obvious but interesting insight is that nobody’s trying to warm the climate (…). It’s a sort of an unintended consequence of the visible manifestation of the life that our minds have created.” “It is the manifestation of generations of exploitative mental habits that have almost inevitably got us to this point”. Most noted that it is the result of a “model of economic development, which has had no regard for the impact on nature, but also really no regard for the impact on human beings (…) treating them (…) just as a factor of production”.

Accordingly, several highlighted that “we have been disconnecting ourselves for centuries now”, and “the climate crisis stems from this disconnected relationship that we have as people with nature in a broad sense: with our own bodies, with the people around us, with the ecosystems we are part of”. Respondents who have been working on climate change mitigation and adaptation for a long time reported that “if you look at those [technological] solutions, you can very quickly see their own limitations in addressing the issue, in the sense that the issue is so much bigger (…). If you don’t start with the mind and this issue of disconnection from nature, you remain in the same type of thinking that you had before”, which led to climate change in the first place.

Several respondents described climate change thus as a symptom of an inner crisis, or as a relationship crisis, which is intrinsically connected to other societal challenges, such as social injustice and political conflict. It was stated that “humans are naturally kind and concerned for others, (…) other living things and the environment. But we live in a society that generally rewards and celebrates the worst in human society about being selfish, self-interested, putting yourself first, doing what’s best for you”. In other words, “our minds have become greedy and (…) acquisitive, and (…) this is reflected outside in an endless pursuit of material goods and possessions”, in our “values, expectations of life, of what success looks like”.

Respondents also touched upon the alienating impact of our contemporary digital economy: “The commercial world that we live in does that through advertisements, as does social media. Attention is the new gold (…) They are forever trying to get our attention to buy this and buy that”. It was argued that it is this “distraction, that lack of connection, which has made us worship money and to not value the things that really connect us”.

#### The mind—a barrier for adequate climate action

Respondents also described how the mind creates barriers to necessary change and action-taking. This was associated with habits of mind, including cognitive bias, default “autopilot” mode and threat responses.

In-group bias (or us-versus-them thinking) was most frequently mentioned, not only in relation to other people, but also other parts of our ecosystem. Cognitive bias can be understood as a systematic “error” in thinking that occurs when we process and interpret information (Kahneman 2011; Stern 2018). It is often a result of our mind’s attempt to simplify information processing and to maintain an energy-saving autopilot mode, thus affecting the decisions and actions that we take (ibid). As one respondent explained, we tend to put “all of nature [in] the out-group, and studies of the brain [show that] you stop having compassion for something you put in the outgroup. So whether it’s a person you’ve deemed in a different racial group or a species that isn’t your species, you treat them just like they’re in the way and they’re just incidental… and so we treat Earth like a trash can”.

Others mentioned that cognitive distortions that make changing habits challenging are (i) polarised thinking (*polarisation effect*, *confirmation bias*), (ii) short-term thinking (*hyperbolic discounting bias*) and (iii) a tendency to blame or rely on others, and not take responsibility for action (*bystander effect*), all leading to a lack of agency and care. As a respondent described, “if your horizons are in your local area [in-group], you feel utterly powerless against the magnitude of the problem (…). You get a number of reactions to that. One is to blame others for it”, or “you deny the problem”. Both cases arise because “there is no feeling that you can make that change, that you can make that difference”, and there is “little drive to support others in doing so”.

The fight-flight-freeze response was said to reinforce such aspects. The latter is our mind’s natural reaction to perceived threats and can be induced by individual, societal and environmental factors. “When we are in a fight-flight-freeze kind of mindset, (…) [it] can make us less empathetic. It can make us more prone to extremist views, more prone to pronounced in-group bias and them-and-us dynamics, all of which reduces the political space for collective action on shared problems, above all, climate change”.

The issue of denial was also frequently mentioned. “The climate crisis is the ultimate existential crisis. (…) We repress, or we grow”. Respondents noted that “people don’t like to be anxious, (…) but the avoidance of anxiety leads to denial (…) that leads to [increased anxiety or] other mental distress like panic disorder”. “The real issue is that the world is in a great deterioration that we all want to try to deny, and sadly in the denial then we do business as usual, and we not only reinforce the problem, we make the problem irreversible”.

#### The vicious cycle of mind and climate change

The above findings show that the relationship between mind and climate change is not perceived as linear, but complex and entangled. Moreover, respondents’ answers indicate how interactions between mind and climate change form feedback loops that degrade personal and planetary wellbeing.

In its simplest form, this “vicious cycle logic” manifests in the following ways: “The state of the climate impacts on our inner lives when we see wildfires or floods or whatever it may be, or just news reports about how bad things are going to be in the future, that activates anxiety”. As a consequence, reflecting dominant social paradigms “We may go shopping to make us feel better. It’s kind of almost like the most universal modern ritual. And obviously, that has direct implications for unsustainable consumption”, which in turn drives climate change. The references to self-reinforcing interactions include many facets that ultimately all translate into deteriorating mental health, climate change and unsustainable responses at individual and collective levels:

*Climate change ⇒ mind as victim ⇒ mind as barrier (e.g. avoidance) ⇒ mind as a victim (e.g. increased anxiety) ⇒ mind as a root cause (e.g. unsustainable coping) ⇒ climate change ⇒ *etc*.*: Climate change-related uncertainty creates anxiety, which we commonly deal with through avoidance that further increases anxiety and unsustainable coping mechanisms in the longer term. “There’s… the inability to look at it. The anxiety that is engendered as a result of that creates a little loop that becomes self-reinforcing and gets worse”.

*Climate change ⇒ mind as barrier (e.g. threat response) ⇒ mind as a barrier (e.g. reduced empathy, polarisation) ⇒ mind as a driver (e.g. individualism) ⇒ climate change ⇒ *etc*.*: Stress and fight-flight-freeze responses to perceived threats reduce empathy and compassion and foster in-group bias and polarisation, impeding social cohesion and collective action needed to address climate change, whilst fostering unsustainable coping and habits that spur it (see Sect. 3.1.3 for related citations). The latter is reinforced by long-term stress leading to reduced self-refection and creativity.

*Mind as a driver (mindset of separation, materialism, individualism) ⇒ mind as a victim (e.g. fear) and barrier (e.g. cognitive bias, attention deficit) ⇒ mind as a victim (e.g. anxiety) and driver (e.g. reinforcing paradigm) ⇒ *etc*.*: The social paradigms and associated mindsets that are at the root of climate change undermine wellbeing, fostering fears and habits of mind that, in turn, keep those paradigms and mindsets alive. For example, after a certain level, economic growth (and associated materialism and individualism) undermines wellbeing, for instance, through creating socialised fears (based on internalised cultural messages of separation) that increase anxiety and foster cognitive biases. The latter is, in turn, influenced by problems with attention, which are both filtered and influenced by our social values and paradigms, our “attention economy”. This is illustrated by how “social media weaponizes our own anxieties against us (…) and triggers us to see the world in them-and-us terms”.

In sum, “there are feedback loops between the state of the world and our states of mind, which we need to get better at both recognising and acting on”. In response to such insights, “there is a growing sense that we’re on the wrong track (…) by being so fixated, for example, on GDP growth (…). It’s not even working on its own terms. And, at the same time, it’s utterly destroying the planet”.

### Links to current policy approaches

Whilst our results reveal a lack of climate policy approaches that integrate inner and outer dimensions of climate change (Sect. 3.2.1), there are isolated advances and individual pioneers who challenge the current institutional and political landscape (Sects. 3.2.2 and 3.2.3).

#### Lack of integrated approaches—fuelling the vicious cycle of mind and climate change

As described in more detail below, current policymaking processes and the resultant approaches fail to address the above-described intertwined nature of mind and climate change. In addition, they reinforce underlying systems, mechanisms and associated individual and collective mindsets, thus further fuelling the vicious cycle of mind and climate change.

All respondents noted that current policy approaches are characterised by a “divorce between inner and outer”. At the same time, there is increasing interest in addressing the inner dimension of climate change because current approaches have been “without success”. One participant observed, “you see the struggle in [the IPCC] Working Group 3 where they try to go to the next step. Why is nothing happening? Why are we every time running into opposition? Why is making change happen so difficult? (…). And you see it also with the European Commission, which is trying in the Green Deal to explicitly talk about actors of change (…). However, it is often only talking”. There was a general agreement among respondents that concrete changes are rare and tend to be instrumental in nature, with policymakers “trying to understand, essentially, how you nudge people towards being able to do things that contribute to emissions reduction”. As expressed by an EU decision-maker, “we don’t get much more beyond these very small nudges (…). And you see people jumping on it, oh, yeah, let’s discuss nudging now for the next 20 years (…) come on (…). This should not be another delaying tactic!” All interviewees expressed the perception that we are only just starting to understand the broader linkages between mind and climate change and the role of inner human qualities/capacities in this context.

Many respondents remarked that in current policymaking, there is a clear denial of the entangled nature of mind and climate change and even a resistance towards its consideration. This resistance was described as an expression of our current dominant social paradigm, where low priority is given to climate change, inner human dimensions and even less to their integration. Consequently, “environmental issues are a bonus that you can have when everything else is going well”, and existing climate policy “focuses on the outer—technology, industrialisation, economic growth (…). They want to fix the problem with more technology, more renewable energy, more solar power, more nuclear power, more wind power (…). But you cannot solve the problem with the same minds or mindsets that created the problem in the first place. (…). Science and technology have been the instruments of producing industrialisation, industrial revolution and materialism and ever-increasing economic growth. And now we want to solve that problem by another kind of technology”.

The divorce between inner and outer aspects is also reflected in the process of policymaking and the qualities/capacities that are considered necessary to develop it. Leadership is said to focus on “external”, professional skills in a system where “emotion is stripped out of it. (…). You immediately get into a discussion on instruments (…), instead of the intrinsic motivation of why are we doing this?” Side effects of the lack of integrative approaches are also seen at an individual level. As one respondent observed, “[we have had] 50 years of amazing innovation in activism—the birth of modern feminism, environmentalism, so much other stuff besides—but often without the necessary inner work. And so you get what we have now, what we see all around us (…): chronic burnout”.

#### Isolated advances challenge current approaches

Despite the general lack of integrated approaches, there are some isolated advances. They largely involve changes regarding policy institutions’ internal management and capacity development for staff, with a few external efforts.

Several interviewees noted a slow convergence between the professional fields of health (wellbeing) and environment (sustainability, climate change) over the past 10 years. Within the European Commission (EC), national governments and other policy institutions, mental health and wellbeing are for instance increasingly considered in the context of capacity development and leadership programmes, counselling and working environment regulations. Whilst such initiatives are generally not climate-specific, there are increasing overlaps, illustrated by recent climate leadership programmes within the EC and national governments, and counselling for citizen climate assembly members. The reasons are manifold. As described by a parliamentarian, “if people have mental resources, if they have wellbeing, then they have also more resources to work against the climate crisis and empathy towards other people (…) less aggression, less racism, less stereotypes, and more resources to work in a positive way towards the planet. This is why I push for such initiatives”. Human resource departments are often seen as an entry point for change. At the same time, “there’s still very much a focus on individual wellbeing, as if it’s almost like a medical problem. (…) There’s so much more to that. But it is a good way in, (…) to start [linking inner and outer transformation]”.

The increased consideration of climate change issues in capacity development and wellbeing-related initiatives has also highlighted the question of what inner qualities/capacities are needed to support transformation towards sustainability. Some leadership courses have been linked to research on this issue, but broader systematic follow-up for improved policy development was said to be lacking. At the same time, there has been increasing support for research on the effects of climate change on wellbeing, as a first step to direct future policy. Examples are surveys by the European Commission (EC) and national governments on climate anxiety and attempts to identify what lifestyle changes children would be willing to accept. Reported results showed that up to 80% of young people experience climate anxiety.

There are only a few efforts to create integrative policies and on-the-ground projects. Exceptions include: (i) new approaches for environmental campaigning communications (e.g. considering eco-psychology or art-based approaches), (ii) the creation of mechanisms and regulations for increasing people’s opportunities to reflect and contribute in a meaningful way (e.g. citizen climate cafés) and (iii) the integration of qualities of mind in political party values and national performance frameworks (e.g. emphasising kindness versus economic growth). The identified interventions are, however, sporadic, piecemeal and driven by individuals. An overall vision, structures and mechanisms for systematic consideration of the intertwined nature of mind and climate change is missing.

#### Individual pioneers: between political marginalisation and concealed endurance

Individual policy pioneers have been a key in making progress in including inner dimensions in climate work. At the same time, they often feel marginalised. As a result, they apply various strategies to achieve change and sustain their efforts, notably, concealed policy integration, internal and external bottom-up work and communities of practice. In parallel, many engage in inner development to increase their personal resilience, which, in turn, has reinforced their climate engagement.

The pioneers who spur the integration of mind and climate change-related issues tend to come either from the field of health, wellbeing and inner development, on the one hand, or sustainability and climate change, on the other. They challenge current paradigms and the associated systems, structures and mechanisms that are currently in place. As described by a former UN official, “I’ve seen individual bright spots of particular people who are able to cut through, but I haven’t seen institutional structures that create that [support for integrating inner and outer dimensions] to be honest”.^4^ Pioneers thus experience many barriers, marginalisation and related stress. For some, it has led to them resigning from their jobs to create, or work for, other organisations of influence. “The whole human dimension was missing; (…) the process was continuing, as long as you were finding technical solutions to problems (…) financial mechanisms (…). This logic didn’t make any sense (…). I decided to leave to do something meaningful”.

Most decide to continue with their work but in a somewhat concealed way. “My colleague always tries to put in things. And it’s always taken out (…). But he’s kind of, ‘yeah, but eventually maybe somebody will miss it and it goes through’. He’s insisting, insisting”. Since policy change is often blocked, advocates also engage in bottom-up initiatives to drive change upwards (e.g. through internal petitions). In addition, many actively engage in (creating or linking to) internal and external support groups. “The sense of being on your own, and doing that, is pretty overwhelming, whereas reaching out to (…) like-minded individuals (…) can give both enormous strength to allow one both to feel the grief, but then to find the resources to respond to it and try to mobilise and campaign against it”.

Several noted that they had begun to engage in practices such as self-awareness, mindfulness and compassion meditation. All but one interviewee described how such practices had, over the years, changed their work, by increasing their circle of identity, care and responsibility for addressing climate change. As described by one interviewee working in the health sector, “It’s changed the focus of my work, to be about the climate crisis and about social justice”. An interviewee from the climate field stated that “it was important to go into this inner dimension of the crisis. (…) It was some kind of no return point, like I couldn’t go back to doing the work the way it was done before, not to consider that [inner dimension] or to see it as an aside from the important work. (…) And then everything changed. Everything changed in how I interacted with institutions, people and what I wanted to work on”. Another policymaker explained how it influenced the conviction “that I have more to give, and I can give it on a bigger scale”.

### Future visions—pathways towards sustainability

Our results show that increasing climate resilience and responsiveness in policymaking and practice requires addressing current barriers and tapping into existing drivers for change (Sect. 3.3.1), supporting transformative qualities/capacities as a gateway for change (Sect. 3.3.2) and linking this approach to other measures to create systems, structures and mechanisms that allow us to move from vicious to virtuous cycles of mind and climate change (Sect. 3.3.3).

#### Contextual barriers and drivers

Policymakers expressed that key barriers for change are the dominant social paradigm and mindset (materialism, consumerism, economic growth) and related political and societal aspects such as power structures, priorities, language, media and digital technology. At the same time, the very same aspects can support change if they are designed to challenge dominant paradigms and mindsets and open up opportunities for new sustainability pathways or narratives to emerge. In this context, younger generations, and societal crises, such as climate anxiety, were mentioned as potential drivers.

The fact that our dominant social paradigm underlies both climate change and current approaches to addressing it was said to make change especially difficult, because addressing the root cause of climate change means questioning current policymaking structures, mechanisms and the ideas that our society is based on. “The solution to the challenge is to draw [our social paradigm or mindset] out of the subconscious and make it visible, which will then revert back to changing our behaviour in relation to it. And that’s proved very hard to do. People have proved very resistant to dragging [that] out and making [it] visible. So how that happens in a manner that doesn’t make people feel threatened or challenged but enables them to feel empowered as part of that process is really important”. This “requires enormous leadership to show people that they could be happy with a different kind of lifestyle, and that the current model is not meeting their real needs, including emotional needs, social needs and fulfilment”.

Policymakers’ current contexts make this challenging. One respondent explained that “organisations [generally] suffer from power games played at the top and powerlessness at lower levels”, but when it comes to change, “the most powerless people are [often] the ones you find at the very top of organisations, because they got there by being totally part of the system”. In addition, “policy is developed by powerful political lobbies”. For instance, “The pharmaceutical industry is worth 1.3 trillion dollars a year, and they spend 20 billion a year on government and public relations and trying to influence decision-makers. And that’s just the pharmaceutical industry. When you look at the oil and gas industry, you’ve got billions, if not hundreds of billions of pounds spent trying to make decision-makers bend this way or bend that way”.

Media and digital technology are also seen as influential forces in this context. Whilst they can be a reflection of the dominant social paradigm and have negative impacts on individual and collective minds, several respondents mentioned that they could also stimulate change. “When industrialisation took away from people the need for much physical labour, then a kind of culture for physical exercise appeared. (…). If we see now that the digital devices have changed how our senses are stimulated (…) then there should also be a counterforce, maybe a kind of mental training of the mind. How are we able to cope with the amount of pressure that is put on our attention? (…) Now we have to deal more with the attention and the capabilities of the mind”. In this context, concepts and professional language are other examples that can be both barriers to and drivers for change. As of today, they do not give importance to inner human dimensions and individuals as agents of change.

Finally, most interviewees mentioned youth movements as an important driver and saw “a way in, to actually give a [stronger] voice to these next generations”. The exponential increase of climate anxiety and the COVID-19 pandemic were also seen as potential drivers for accelerating collective awareness and pushing politicians to act.

#### The mind—nourishing transformative qualities/capacities as a gateway for individual and planetary wellbeing

Many respondents spoke of the urgent need for a courageous move from the current mainstream ontology of separateness and self-centeredness towards one of entanglement and connectedness. Nourishing inner qualities/capacities was, in this context, often seen as a gateway that is needed to “transcend a paradigm that you are operating inside”. As one former UN official explained, “you have to realise at quite a fundamental level that you’re constructing your world through (…) the filter that you put everything through. So softening that requires you to have a tiny bit of space between phenomena and mental reaction”.

Accordingly, the most frequently mentioned transformative qualities/capacities related to aspects of awareness, such as self-awareness, self-reflection, presence, attention, acceptance, openness and emotional regulation. Others were associated with relational aspects such as compassion and empathy, and self, other, and human-nature connectedness. Finally, perspective-taking, intrinsic value orientation and qualities that can instil agency for action-taking were also noted. Mindfulness and compassion training were associated with most of these qualities/capacities (see Suppl. Material).

Agentic qualities included courage, optimism and hope. Together with the awareness-based and relational capacities mentioned above, they were said to be key to facilitate action, despite uncertain outcomes. This requires moving from an ontology of separateness to one of connectedness. It was described as a way “to break a mental habit of ignoring something and then feeling anxious about it, and then ignoring it more and feeling anxious about it. (…). Instead of that sort of rejection and aversion, it is something that feels like a great shared endeavour that is inspiring”. “It can flip people’s habits from a sense of powerlessness (…) to quite a different sense of participation and meaning. In that there’s one particular dynamic that we’ve gotten wrong (…), that’s the relationship between meaning and control. In most areas of human life, meaning has nothing to do with control (…). In climate change, we say unless you have control, there’s no meaning. (…) Why are you bothering to try to do X, Y and Z? (…) We need to move beyond that and start realising [that] taking action to protect the planet, to protect future generations (…) is some of the most meaningful work that we can do at this moment, at whatever level is appropriate for us, even though, perhaps especially because, we can’t control the outcome”. Ultimately, “how we deal with the global challenges in this decisive decade will be determined by who we are, and how we’re able to show up to that moment”.

As pointed out by one policymaker, the interaction between mind and climate change thus needs to be better considered in two key areas: First, within the content and social impacts of policies, regarding the integration of inner and outer worlds and how qualities of mind are nourished or hampered. Second, within the policymaking process itself and the inner qualities/capacities that are needed to make it transformative. As illustrated by a former UN member of staff, “treaties are negotiated by people, not by countries, and progress is determined far more by individual characteristics, individual styles, individual relationships than is evident at all”. Regarding both areas, support for capacity development, experiential learning and creating safe spaces for reflection, dialogue and contemplation were mentioned as important measures.

#### From vicious to virtuous cycles of mind and climate change: learning from integrated measures and approaches

Moving from vicious to virtuous cycles of mind and climate change was said to require the systematic consideration of mind across all sectors of work. This involves adapting current organisational structures, mechanisms and policies to create the conditions for a new, more sustainable narrative to emerge.

Lessons from existing efforts and policy integration from related fields provide input as to how this can be achieved in practice (see Sect. 3.2.2 and Table 1 for illustrative examples). Systematising such lessons was seen as important to provide policymakers with what could be called accessible “nuggets” to act on inner climate impacts such as eco-anxiety and overwhelm and, at the same time, address the drivers and root causes of the crisis. As described in the previous sections, this requires direct (e.g. capacity development) and indirect (structural) interventions that can be supported by policy. The latter is crucial to scale up current efforts and link them in a way that ensures the comprehensive and systematic consideration of the mind across personal, collective, organisational and system levels. In this context, some policymakers noted that insights from policy integration could help to provide a roadmap.

**Table 1:** Illustrative examples of suggested measures/nuggets for addressing climate change, easing climate anxiety and empowering agents of change through the comprehensive integration/mainstreaming of issues of mind and associated transformative qualities/capabilities in policy and action

Overview of policy mainstreaming strategies	Illustrative examples of related policy interventions and associated project measures
O**rganisational, internal and inter-organisational mainstreaming** This involves three strategies that focus on the organisational level: i) The modification of the organisation’s/department’s policy, corpus of legislation, management and working structures, along with project instruments in the policy cycle to ensure the consideration and institutionalisation of aspects of MIND* in climate-related sector work; ii) The modification of the organisation’s/department’s way of operating and its internal policies to reduce its own risk (related to aspects of MIND*) and ensure its continuous functioning in a context of increasing climate change and associated impacts; iii) The promotion of collaboration between the organisation/department and other stakeholders (international, regional and local governmental, and civil society) to generate shared knowledge, develop competence and take joint actions to advance the integration of MIND* in climate-related work.	The revision of regional, national or local performance frameworks by integrating values and/or transformative qualities/capacities (such as kindness or compassion) as explicit aims/criteria (versus economic growth or a pure focus on CO_2_ reductions). The revision of educational policies and national teacher training standards to make the below-mentioned changes for sustainability education a legal right for all citizens. The provision of transformative spaces for improving climate negotiations/collaboration in the form of safe ‘containers’ for self-reflection and enquiry into the role of mind in climate change and sustainability, to challenge current, unsustainable systems and paradigms and create the conditions for emergence. The revision of organisations’ mission statements regarding sustainability to support the idea that individual and planetary wellbeing are intrinsically related, and central to the organisation’s internal and external engagements/portfolio. The revision of project planning and management processes and tools, such as results-based/logical framework approaches, by using aspects of mind (e.g. intrinsic, universal values and capacities) as the underpinning factor for defining climate-related inputs, outcomes, outputs and impacts. The revision of related monitoring and evaluation tools, by also considering the aspect of emergence. Change in environmental campaigning to avoid triggering fight-flight-freeze reactions and polarisation, and instead to come from a point of shared humanity and universal values.
**Educational mainstreaming** This strategy links to education more broadly and involves the support for a conceptual shift (individual and collective/cultural) in the philosophy that drives climate-related education and stewardship. The aim is that considerations of MIND* become inherent to all sectors and spheres of activities that are relevant to address climate change.	The integration of climate change considerations into education across disciplines/sectors (school, professional education, leadership and adult development) by: i) adopting an integrated approach that also addresses the underlying root causes/mindsets, and ii) putting increased emphasis on vertical (as opposed to horizontal) learning. This involves strengthening transformative qualities/capacities to support agency and equitable transformation. The creation of educational platforms for climate change education (e.g. exhibitions, professional networks, communities of practice) to inspire or co-create new sustainability imaginaries/paradigms. The provision of transformative spaces that can nurture fields of change (see also above).
**Add-on and programmatic mainstreaming** This strategy focuses on the local level and involves the integration of aspects of MIND* into the organisation’s/department’s core, on-the-ground projects.	The creation of citizen climate cafés, local climate councils and/or counselling where citizens can express their emotions related to climate change, address climate anxiety and have the opportunity to engage in meaningful public–private cooperation projects. The creation of local knowledge platforms for the recognition and inclusion of local, traditional and/or indigenous knowledge systems, perspectives and approaches in decision-making to challenge current, unsustainable approaches/paradigms/narratives. The improvement of project-related climate communication and environmental campaigning in a way that links climate change to other societal crises, and addresses related internal dimensions (e.g. by sourcing intrinsic values [in oneself and those addressed] and/or supporting agency, hope and optimism versus climate anxiety and denial).

* In climate policy mainstreaming, this relates to the integration of climate objectives into sector work and policy. Here, it is the mutual integration of climate objectives and aspects of mind. The challenge lies in the fact that the integration of climate objectives has, so far, not been achieved. In practice, this means that climate change is in many organisations/departments still addressed as almost separate from relevant sector work and deeper ecological crises. Note that the terms institution(al) and organisation(al) are used interchangeably in this framework/article.

The motivation for policy integration/mainstreaming originates from the need to change the dominant paradigm. It consists of a set of strategic activities and has been used in the past in various fields to integrate cross-cutting issues such as gender equality, health/wellbeing, environment and climate. Mainstreaming was described by interviewees as improving existing systems, mechanisms and structures by embedding new aspects and practices “that will sort of become more and more embedded as the bedrock of something bigger”; over time, they become “a manifestation of the underlying culture”.

Moving from vicious to virtuous cycles of mind and climate change can thus be described as a two-way process. On the one hand, “it’s the underlying culture that needs to evolve, and the institution-building follows culture”. On the other hand, the institutional and policy landscape can be designed to support the emergence of certain cultural expressions and narratives. The mainstreaming of sustainability and wellbeing considerations into education was, in this context, seen as an important case to learn from. The “whole school” approach to learning for sustainability ensures transformation by nourishing inner aspects through capacity development that includes experience-based social, emotional and ethical learning, which is enabled through systems, mechanisms and structures that together create the conditions for a new, more sustainable narrative to emerge. Measures include, for instance, the revision of school curricula, the integration of caring for the environment in the school’s culture/values, the involvement of the surrounding community in local projects and the revision of related national regulations and policies. The latter could ensure that learning for sustainability is made a legal entitlement for every school child, including outdoor learning as a regular and progressive part of the education. In addition, “being able to teach about sustainable development, and not just the knowledge of it, but the values and attitudes that go around it” could be integrated into national teacher training standards.

Mainstreaming wellbeing and mindfulness into public policy regarding health, education and the workplace was mentioned as another example to learn from. Related initiatives focused mainly on interested individuals (capacity development) and the creation of associated communities of practice. The need to support a community of practice or a so-called field was mentioned by many respondents as key for challenging current paradigms and mindsets. “What you’re doing is creating both a sense of group identity, but also the networks for intensive learning and knowledge sharing, and then also you’re creating an ecosystem that funders can begin to support (…) starting with some pilot projects”. Another lesson was that research programmes should be developed to create a better scientific foundation and build arguments for policy interventions as a first step within prevailing paradigms (e.g. financial returns). In addition, an emphasis on certain policy fields and strategic measures (e.g. resource allocation, research, training) was suggested as a way to address immediate needs and, at the same time, support long-term transformation. Finally, both content and approaches need to be revised in relation to specific sectors or contexts and targeted resources and roadmaps be developed.

## Discussion and conclusions: policymaking for a more conscious and caring society

**Fig. 1:**
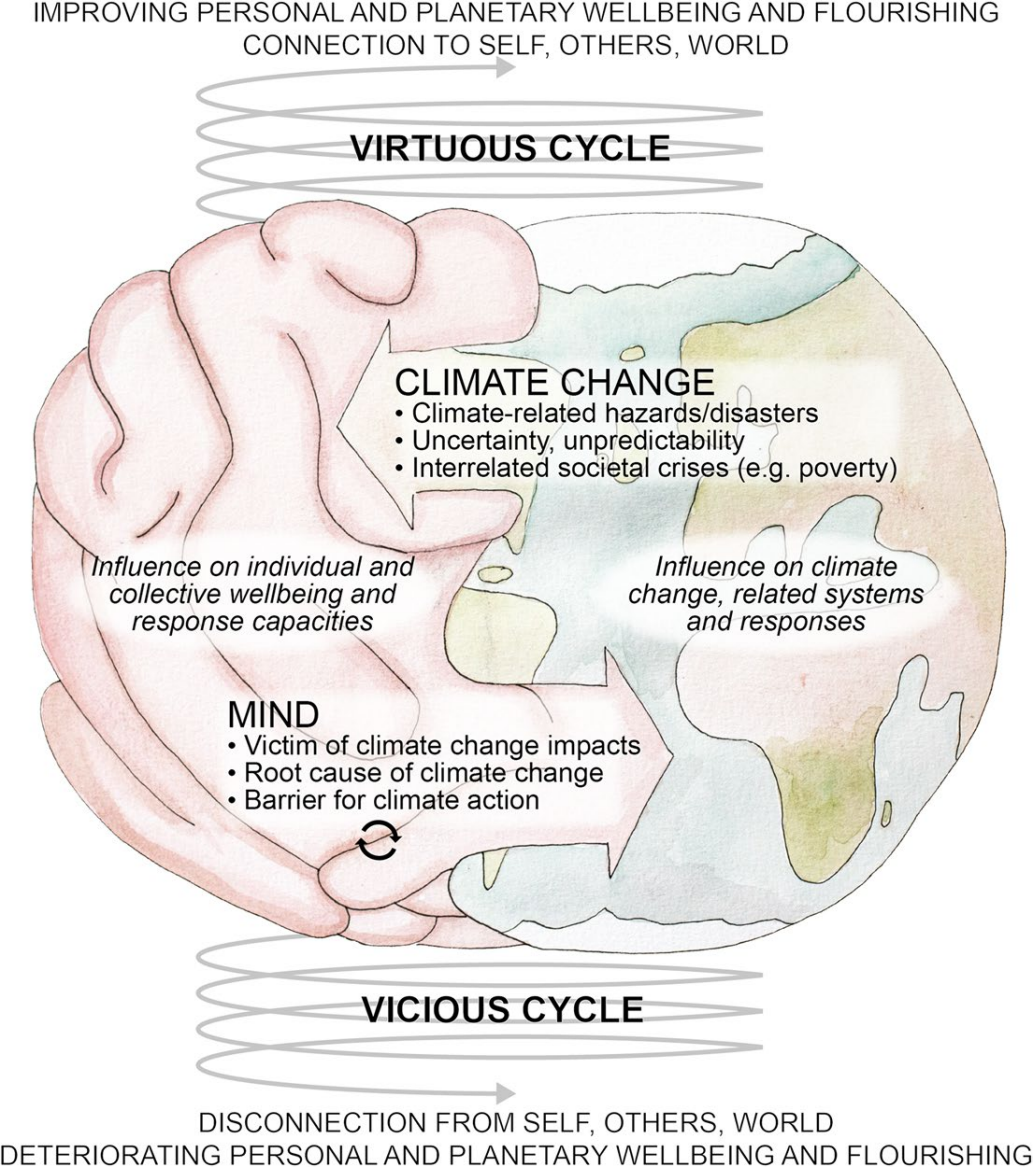
Illustration of the entangled nature of mind and climate change and its potential role in policymaking and practice to foster personal and planetary wellbeing. Illustration: Emma Li Johansson

This study demonstrates how the entangled nature of mind and climate change is perceived and addressed in current climate policymaking and practice. Policymakers and advisors report a trend towards greater consideration of the issue. However, this is tightly restricted to a desire to address the rising impact of climate anxiety. It is almost absent from other policy initiatives and even appears to be actively resisted in institutions. This relates to the fact that current approaches operate within the same collective mindset and paradigm that underlie the climate crisis and fuel the identified vicious cycle between mind and climate change, which, ultimately, can degrade individual and planetary wellbeing (Fig. 1). As climate disruption deepens, and the impact on wellbeing and related perceptions become more severe, feedback will become stronger.

Our results support those of other studies that show that scientific data, although undoubtedly vital for alerting our rational mind to the existence of a threat, does not galvanise us into action (Clayton 2019; Frisk and Kelli 2011; Grušovnik 2012; Suldovsky 2017). They show that we tend to act on our feelings and that these are, in turn, so entrenched in our dual, disconnected lives that they obscure intrinsic human values and the connection that is crucial to address the climate crisis (Clayton and Manning 2018; Petersen et al. 2019; Wamsler et al. 2021). Our results show that they are embedded in our modern, dualistic worldview, which also translates into reported disciplinary specialisation, professional silos, the lack of importance given to individuals as agents of change and the fact that current institutions and policymaking, and the way people think about them, is predicated on a rigid separation between inner and outer. In addition, as years of scientific research in the field of environmental psychology have also shown, our minds find it difficult to deal with long-term issues and delayed impacts, with climate change and other societal crises such as the COVID-19 pandemic, being a recent and dreadful demonstration of this (APA 2010; Clayton 2019; Dörner 1997; Guerriero et al. 2020).

If we cannot find a way to address the identified vicious cycle of mind and climate change, feelings of disconnection, powerlessness and alienation, which other research has also increasingly identified as a barrier to appropriate action,^5^ will grow (Clayton and Manning 2018; Palinkas and Wong 2020). In addition, other research also shows that deteriorating mental health and increasing fear and threat responses will slide us further into them-and-us dynamics—polarisation, othering, hate, conflict and breakdown in collaboration and climate action (Corner et al. 2012; Doherty and Clayton 2011; Weber et al. 2021). An increase in such dynamics is a growing concern and a key challenge in sustainability and climate work worldwide (Cianconi et al. 2020; Leichenko and O’Brien 2020; Palinkas and Wong 2020).

Against this background, our study shows that the way current climate policy is designed and developed increasingly appears to be deficient. The remedy is to use targeted measures to develop more integrative approaches (Table 2), which have also been called for by the recent IPCC reports (IPCC 2022a,b). Without such efforts, the system will continue to beat the individual. As our results show, when policy pioneers and leaders attempt to bring aspects of mind and inner transformation into their current work, they are often marginalised, suppressed and excluded, and some even decide to leave their organisations to have more impact. This is in stark contrast to the idea that “a core goal of public policy should be to facilitate the development of institutions that bring out the best in humans”, as stated by Elinor Ostrom when she received her Nobel Prize in Economics (Ostrom, 2009). To address related gaps, we thus need structures and practices that can protect risk-takers and support personal development (Sharma 2017), together with a new way to structure and run organisations, and collaborate and question every aspect of policy and project management (Jacob et al. 2021; Laloux 2014).^6^

**Table 2:** Policy recommendations for governments, public institutions, and private sector

Focus area	Recommendation	Related considerations and examples*
Targeted sustainability and climate work – international, national and local levels	Policy support for the systematic consideration of the role of mind and transformative qualities/capacities in the development and implementation of sustainability and climate-related policy.	Modification of existing regulations, processes and structures at all policy levels and across all sectors, e.g. how the issue of climate change is portrayed and communicated, the goals that are pursued, the measures that are promoted, how they are implemented, and the performance targets that are applied. Special units and staff mandated to support related processes.
Education – primary and secondary school, university, and adult development	Policy support for improved sustainability education, which balances knowledge development and professional skills with the cultivation of inner human qualities/capacities that underpin individual and societal flourishing and sustainability.	Legislation that ensures that education regarding ecological concerns and their important implications for future generations is balanced with the development of the inner qualities/capacities that are required to cope with the emotional cost and respond appropriately and is offered across all disciplines and sectors.
Professional development and climate leadership – all levels and sectors	Support for improved climate leadership, assisting leaders in their understanding and cultivation of inner qualities/capacities and aspects of mind that underpin comprehensive climate action at individual, collective and system levels.	Funding of leadership training that helps to develop an understanding of human biases and common psychological defence mechanisms to threatening messages, so that leaders’ climate strategies become better-informed and more likely to overcome resistance to challenging climate information and polarisation.
Health care and health promotion – international, national and local levels	Policy support for addressing eco-anxiety, -grief, and -depression for citizens, particularly the young.	Legislation to support professional training for better-recognising and addressing the mental health impacts of the climate crisis. Measures for healthy child and adult capacity development in the context of climate change and digital technology; support of inner capacities to help protect people from the negative impacts.
Research	Increased funding for critical, inter- and transdisciplinary research on the role, development and implementation of more integrative approaches that link inner and outer dimensions of climate change to support sustainability and transformation across individual, collective and system levels.	Programs for investigating the vicious cycle of climate change, threat response and trauma, poor mental health, worldviews of separateness and disengagement, and the development and testing of methods and approaches that may interrupt and reverse this cycle. Support for research into how certain methods, inner qualities/capacities and leverage points relate to the United Nations 17 Sustainable Development Goals.

*See also Table 1 for further examples and measures

Importantly, our results show that moving from a vicious to a virtuous cycle requires us to question the very foundations of our self-view, society and current policy approaches to sustainability if we want to move towards a more conscious and caring society (Feola et al. 2021; Marion Suiseeya et al. 2021; Wamsler et al. 2021). This requires the systematic integration of considerations of mind in *all* sector work and *across* personal, collective, organisational and system levels to support the emergence of new approaches (Wamsler et al., 2021). In turn, this entails implementing accessible “nuggets” (and not nudges) at organisational and project levels, and creating communities of practice, whilst systematically integrating aspects of mind into all sectoral policies, processes and programmes. In addition, our study shows that education and capacity development for decision-makers and the general public is a key to increasing knowledge on how minds shape climate change, systems and behaviour and nourish transformative qualities and capacities that are required to turn the vicious cycle into a virtuous cycle. These results and associated recommendations support and build on existing work in the field of education (Brundiers et al. 2021; Frank 2021; Giangrande et al. 2019) and at the same time provide new knowledge and examples that form a foundation and a roadmap for such endeavours.

We conclude with a call for increased strategic policy engagement, research, resource allocation and education for addressing the role of the mind in climate policymaking and practice. Our results provide a comprehensive systematisation and illustration of how our minds are driving the crisis and obstructing responses across individual, collective and policy levels, which is crucial for developing more integrative approaches and action. Given the centrality of the mind in underpinning the climate crisis, and its relative absence from mainstream approaches, this should also be a high priority for further critical research which involves processes that induce reflexivity regarding both researchers’ and participants’ perspectives and mindsets (Adger et al., 2013; Hulme 2009; Ives et al. 2020; O’Brien 2018).

Both research and policy support are needed to reduce resistance within institutions to more integrative sustainability initiatives and provide guidance on how this can best be achieved in different sectors and contexts. Such support is a key for achieving the sustainable development goals and international climate agreements through linking personal and planetary wellbeing. At the same time, we must be aware that the very formulation of some of these texts is part of the problem and, thus, should be questioned, for instance, when they reinforce unsustainable paradigms and mindsets.

## Supplementary Information

Below is the link to the electronic supplementary material.

Supplementary file1 (PDF 1.56 MB)

## Data Availability

Additional data is included as electronic supplementary material.

## Footnotes

1 These issues are commonly also referred to as inner, interior, internal or personal dimensions.

2 Networks, such as climate ambassadors and climate leadership development groups within the EC, national parliaments and governments and policy institutions were used to make contact with policymakers and ask if they would be willing to participate in a survey or interview. In addition, Google Scholar and search terms such as inner capacities/dimensions/transformation, climate change and sustainability were used to identify scholars who have been active in the field in recent years. We contacted these people and reviewed their work in order to identify further study participants.

3 The identified themes and patterns are presented under each subsection in the “Results”. Frameworks and illustrative examples of the identified themes and patterns are included in the Suppl. Material.

4 At the same time, one interviewee noted that in the case of climate agreement negotiators, what they think or value personally is not necessarily what they bring to the table. This is also related to the question of how to move from a focus on individual mindsets to cultural change so that individuals can express their intrinsic values and humanness within current systems and structures without losing their job.

5 It is thus necessary to differentiate between climate inaction and harmful climate action. Climate action tends to refer to technological solutions (e.g. renewable energy, less packaging, more virtual communication) to enable reasonably wealthy segments of society to maintain their standard of living and “feel green”, although those actions are likely to strengthen inequalities and can have major environmental consequences in relation to rare mineral mining and waste management, among others.

6 The role of collective mindsets, both in the organisation and beyond, is in this context crucial so that the pioneers and risk-takers benefit from an *arriere-garde* and can push transformation further.

## References

[CR1] Adger WN, Barnett J, Brown K, Marshall N, O’Brien K (2013). Cultural dimensions of climate change impacts and adaptation. Nat Clim Chang.

[CR2] American Psychological Association. (2010). *Psychology and global climate change: addressing a multi-faceted phenomenon and set of challenges*. https://www.apa.org/science/about/publications/climate-change

[CR3] Bamberg S, Möser G (2007). Twenty years after Hines, Hungerford, and Tomera: a new meta-analysis of psycho-social determinants of pro-environmental behaviour. J Environ Psychol.

[CR4] Braun V, Clarke V (2006). Using thematic analysis in psychology. Qual Res Psychol.

[CR5] Brundiers K, Barth M, Cebrián G, Cohen M, Diaz L, Doucette-Remington S, Dripps W, Habron G, Harré N, Jarchow M, Losch K, Michel J, Mochizuki Y, Rieckmann M, Parnell R, Walker P, Zint M (2021). Key competencies in sustainability in higher education—toward an agreed-upon reference framework. Sustain Sci.

[CR6] Carter DM (2011). Recognizing the role of positive emotions in fostering environmentally responsible behaviors. Ecopsychology.

[CR7] Cianconi P, Betrò S, Janiri L (2020). The impact of climate change on mental health: a systematic descriptive review. Front Psych.

[CR8] Clayton S (2019). Psychology and climate change. Current Biology: CB.

[CR9] Clayton S, Manning C (Eds.). (2018). *Psychology and climate change: human perceptions, impacts, and responses* (1st edition). Academic Press.

[CR10] Conceição P (2020). *Human development report 2020: the next frontier: human development and the Anthropocene*. UNDP. https://hdr.undp.org/content/human-development-report-2020

[CR11] Corner A, Whitmarsh L, Xenias D (2012). Uncertainty, scepticism and attitudes towards climate change: biased assimilation and attitude polarisation. Clim Change.

[CR12] Doherty TJ, Clayton S (2011). The psychological impacts of global climate change. Am Psychol.

[CR13] Dörner D (1997). The logic of failure: recognizing and avoiding error in complex situations.

[CR14] Feola G, Koretskaya O, Moore D (2021). (Un)making in sustainability transformation beyond capitalism. Glob Environ Chang.

[CR15] Figueres C, Rivett-Carnac T (2020) *The future we choose: surviving the climate crisis*. Knopf Publishing Group.

[CR16] Fischer J, Riechers M (2019). A leverage points perspective on sustainability. People and Nature.

[CR17] Frank P (2021). A proposal of personal competencies for sustainable consumption. Int J Sustain High Educ.

[CR18] Frisk E, Kelli L (2011). Educating for sustainability: competencies & practices for transformative action. J Sustain Educ.

[CR19] Giangrande N, White RM, East M, Jackson R, Clarke T, SaloffCoste M, Penha-Lopes G (2019). A competency framework to assess and activate education for sustainable development: addressing the UN Sustainable Development Goals 47 challenge. Sustainability.

[CR20] Göpel M. (2016). *The great mindshift: how a new economic paradigm and sustainability transformations go hand in hand* (1st ed. 2016 edition). Springer.

[CR21] Grasso M, Tàbara JD (2019). Towards a moral compass to guide sustainability transformations in a high-end climate change world. Sustainability.

[CR22] Grušovnik T (2012). Environmental denial: why we fail to change our environmentally damaging practices. Synthesis Philosophica.

[CR23] Guerriero C, Haines A, Pagano M (2020). Health and sustainability in post-pandemic economic policies. Nature Sustainability.

[CR24] Hedlund-de Witt A, de Boer J, Boersema JJ (2014). Exploring inner and outer worlds: a quantitative study of worldviews, environmental attitudes, and sustainable lifestyles. J Environ Psychol.

[CR25] Hulme M (2009). Why we disagree about climate change: understanding controversy, inaction and opportunity.

[CR26] IDG Initiative. (2021). *Inner development goals (IDG): background, method and the IDG framework*. https://static1.squarespace.com/static/600d80b3387b98582a60354a/t/616eb1adbee9380a25085e35/1634644401138/211019_IDG_Report.pdf

[CR27] IPCC. (2022a). *Climate Change 2022a**: impacts, adaptation, and vulnerability. Contribution of Working Group II to the Sixth Assessment Report of the Intergovernmental Panel on Climate Change*. [H.-O. Pörtner, D.C. Roberts, M. Tignor, E.S. Poloczanska, K. Mintenbeck, A. Alegría, M. Craig, S. Langsdorf, S. Löschke, V. Möller, A. Okem, B. Rama (eds.)]. Cambridge University Press. In Press.

[CR28] IPCC. (2022b). *Climate Change 2022b**: mitigation of climate change. Contribution of Working Group III to the Sixth Assessment Report of the Intergovernmental Panel on Climate Change*. [P.R. Shukla, J. Skea, R. Slade, A. Al Khourdajie, R. van Diemen, D. McCollum, M. Pathak, S. Some, P. Vyas, R. Fradera, M. Belkacemi, A. Hasija, G. Lisboa, S. Luz, J. Malley, (eds.)]. Cambridge University Press, Cambridge, UK and New York, NY, USA. doi: 10.1017/9781009157926

[CR29] Ives CD, Freeth R, Fischer J (2020). Inside-out sustainability: the neglect of inner worlds. Ambio.

[CR30] Jacob K, Paulick-Thiel C, Teebken J, Veit S, Singer-Brodowski M (2021). Change from within: exploring transformative literacy in public administrations to foster sustainability transitions. Sustainability.

[CR31] Kahneman D (2011). Thinking, fast and slow.

[CR32] Klöckner CA (2013). A comprehensive model of the psychology of environmental behaviour—a meta-analysis. Glob Environ Chang.

[CR33] Köhler J, Geels FW, Kern F, Markard J, Onsongo E, Wieczorek A, Alkemade F, Avelino F, Bergek A, Boons F, Fünfschilling L, Hess D, Holtz G, Hyysalo S, Jenkins K, Kivimaa P, Martiskainen M, McMeekin A, Mühlemeier MS, … Wells P (2019) An agenda for sustainability transitions research: state of the art and future directions. Environmental Innovation and Societal Transitions, 31, 1–3210.1016/j.eist.2019.01.004

[CR34] Laloux, F. (2014). *Reinventing organizations: a guide to creating organizations inspired by the next stage in human consciousness*. Nelson Parker.

[CR35] Legrand T, Jervoise A, Wamsler C, Dufour C, Bristow J, Bockler J, Cooper K, Corção T, Negowetti N, Oliver T, Schwartz A, Steidle G, Taggart S, Søvold L, Wright J (2022) *Cultivating inner capacities for regenerative food systems: rationale for action*. United Nations Development Programme UNDP.

[CR36] Leichenko R, O’Brien K (2020). Climate and Society: transforming the future.

[CR37] Marion Suiseeya KR, Elhard DK, Paul CJ (2021). Toward a relational approach in global climate governance: exploring the role of trust. Wires Clim Change.

[CR38] Meadows D (1999). *Leverage points: places to intervene in a system*. The Sustainability Institute

[CR39] Nielsen KS, Clayton S, Stern PC, Dietz T, Capstick S, Whitmarsh L (2021). How psychology can help limit climate change. Am Psychol.

[CR40] Nowell LS, Norris JM, White DE, Moules NJ (2017). Thematic analysis: striving to meet the trustworthiness criteria. Int J Qual Methods.

[CR41] O’Brien K (2018). WRONG Is the 1.5°C target possible? Exploring the three spheres of transformation. Curr Opinion Environ Sustain.

[CR42] Ostrom, E. (2009, December 8). *Beyond markets and states: polycentric governance of complex economic systems (Nobel Prize Lecture)*. https://www.nobelprize.org/prizes/economic-sciences/2009/ostrom/lecture/

[CR43] Palinkas LA, Wong M (2020). Global climate change and mental health. Curr Opin Psychol.

[CR44] Parodi O, Tamm K (Eds.). (2018). *Personal sustainability: exploring the far side of sustainable development*. Routledge. 10.4324/9781315159997

[CR45] Petersen E, Fiske AP, Schubert TW (2019). The role of social relational emotions for human-nature connectedness. Front Psychol.

[CR46] Rimanoczy I (2014). A matter of being: developing sustainability-minded leaders. J Manage Glob Sustain.

[CR47] Sharma M (2017). *Radical transformational leadership: strategic action for change agents*. North Atlantic Books.

[CR48] Stern MJ (2018). Cognitive biases and limitations.

[CR49] Suldovsky B (2017). The information deficit model and climate change communication. In Oxford Res Ency Clim Sci.

[CR50] UNFCCC. (2020a). *Conference of the Parties (COP) (2020a**)*. https://unfccc.int/process/bodies/supreme-bodies/conference-of-the-parties-cop

[CR51] UNFCCC. (2020b). *Nationally determined contributions (NDCs): the Paris Agreement and NDCs*. https://unfccc.int/nationally-determined-contributions-ndcs

[CR52] Waddock S (2015). Reflections: intellectual shamans, sensemaking, and memes in large system change. J Chang Manag.

[CR53] Wamsler C (2015). Mainstreaming ecosystem-based adaptation: transformation toward sustainability in urban governance and planning. Ecol Soc.

[CR54] Wamsler C, Osberg G, Osika W, Herndersson H, Mundaca L (2021). Linking internal and external transformation for sustainability and climate action: towards a new research and policy agenda. Glob Environ Chang.

[CR55] Wamsler C, Schäpke N, Fraude C, Stasiak D, Bruhn T, Lawrence M, Schroeder H, Mundaca L (2020). Enabling new mindsets and transformative skills for negotiating and activating climate action: lessons from UNFCCC conferences of the parties. Environ Sci Policy.

[CR56] Weber TJ, Hydock C, Ding W, Gardner M, Jacob P, Mandel N, Sprott DE, Van Steenburg E (2021). Political polarization: challenges, opportunities, and hope for consumer welfare, marketers, and public policy. J Public Policy Mark.

[CR57] Woiwode C, Schäpke N, Bina O, Veciana S, Kunze I, Parodi O, Schweizer-Ries P, Wamsler C (2021). Inner transformation to sustainability as a deep leverage point: fostering new avenues for change through dialogue and reflection. Sustain Sci.

