# Supplementary material for: At the intersection of mind and climate change: integrating inner dimensions of climate change into policymaking and practice
Source: Clim Change. 2022 Jul 15;173(1-2):7. doi: 10.1007/s10584-022-03398-9 (PMC9285190; doi:10.1007/s10584-022-03398-9)
Supplement: Supplementary file 1 — Supplementary file1 (PDF 1.56 MB) [file 10584_2022_3398_MOESM1_ESM.pdf]

6 Supplementary Material

A Highlights

- Policymakers begin to see the mind as a victim and driver of climate change, and a barrier to action.
- Our individual and collective minds/ mindsets are at the root of today's climate crisis.
- Current policy approaches ignore and therefore reinforce the vicious cycle between mind and climate change
- Together, they lead to the deterioration of individual and planetary wellbeing.
- This fact is not reflected in mainstream climate policy and policymaking approaches.
- We identify the aspects that currently hamper and support more integrative policy approaches.
- We provide insights into how future climate policy can better-integrate inner and outer dimensions of change.

B Interview guide

1. I would like to start by asking you, in a broad sense, how you think people's minds, people's inner lives relate to the climate crisis? How do you understand this relationship? What interactions come to mind?
2. Based on the interactions you just described, are there any kinds of inner human qualities or capacities* that you perceive as particularly essential for increasing responsiveness and resilience to the climate crisis, and why? * Refers here to psychological, emotional/cognitive capacities rather than technical and professional skills.
3. If you are familiar with it, how do you see the role of mindfulness and compassion in this context? How do you see their role in climate responsiveness and resilience?
4. On a more personal level, how do you make use of, or intentionally cultivate, inner capacities to support your work? *(If mindfulness is mentioned, follow-up by asking: Since when have you been practicing, and how has it influenced your work, e.g. links to sustainability, climate responsiveness, climate resilience).*
5. What role do inner qualities/ capacities play in your own work? Are they for instance considered in the projects you are involved in, working processes and structures, policies or resource allocation?
6. Do you know of any other examples of how inner qualities/ capacities have been integrated in any policy work to support responsiveness or resilience to the climate crisis?
7. What barriers prevent you or other policy-makers and decision-makers from further considering inner capacities in sustainability and climate work?
8. Finally, I would like to ask you if there is any particular framework that you or your colleagues use for understanding the main drivers of the climate crisis? And if yes, are inner aspects, such as people’s minds considered here?

Thank you for your time! Please let me know if you would recommend any other people who I should contact for this initiative.

C Survey questions

Survey for Policy- and Decision-Makers

1. What is your role?

- Policy- / decision-maker at global level
- Policy- / decision-maker at EU level
- Policy- / decision-maker at national level
- Policy- / decision-maker at regional or local level (e.g. city)
- Other, please specify

2. Which area do you work in primarily?

- Parliament or other legislature
- Public sector
- Private sector
- Non-governmental organisations
- Educational body
- Other, please specify

3. What is your country of residency?

4. Take a moment to reflect on how people's inner lives relate to the climate crisis. How do understand this relationship? What interactions come to mind?

5. What kinds of inner human qualities/ capacities* do you perceive as essential for increasing responsiveness and resilience to the climate crisis, and why? Please list those you find essential and explain your reasoning. * Refers here to psychological, emotional/cognitive capacities rather than technical and professional skills.

6. If you are familiar with mindfulness and compassion practices, how do you see their role in climate responsiveness and resilience?

7. Take a moment to reflect on the role of inner qualities/ capacities in your own work. How could these capacities be integrated to support climate responsiveness and resilience in this context? (e.g. daily activities, projects, communication, strategic aims, working processes and structures, resource allocation, etc.)

8. On a more personal level, how do you make use of, or cultivate, inner qualities/capacities to support your work?

9. Please indicate below if you would recommend any policymaker or any other people who we should contact for this consultation (please include name, affiliation and email address, if possible)

10. Finally, please indicate below if you would be willing to be contacted for any follow-up questions (please include your email address).

Please tick below the "Submit" box to finalise the survey.

Thank you for taking the time to complete this survey - your input is greatly appreciated. Please feel free to forward this survey to any contacts you have with expertise in the area of inner capacities and their relation to the climate crisis.

Survey for Policy-Advisors and Decision-Makers

1. Which best describes your professional role?

- Advocate / campaigner
- Leader / manager
- Academic / researcher
- Innovator / practitioner
- Writer / thinker / communicator
- Other, please specify

2. Which sector do you work in?

- Public sector
- Private sector
- Non-governmental organisations

- Educational body
- Other, please specify

3. What is your country of residency?

4. Take a moment to reflect on how people's inner lives relate to the climate crisis. How do understand this relationship? What interactions come to mind?

5. What kinds of inner human qualities/ capacities* do you perceive as essential for increasing responsiveness and resilience to the climate crisis, and why?
Please list those you find essential and explain your reasoning. * Refers here to psychological, emotional/cognitive capacities rather than technical and professional skills.

6. If you are familiar with mindfulness and compassion practices, how do you see their role in climate responsiveness and resilience?

7. Have you done any research, innovation or communication relevant to the intersection of inner-capacity development and responsiveness or resilience to the climate crisis? If so, please describe your work.

8. What message, insight or recommendation would you like policymakers working on the climate crisis to hear - and what considerations would be important when choosing your language or framing?

9. Please indicate below if you would recommend any policymaker or any other people who we should contact for this consultation (please include name, affiliation and email address, if possible)

10. Finally, please indicate below if you would be willing to be contacted for any follow-up questions (please include your email address).

Please tick below the "Submit" box to finalise the survey.

Thank you for taking the time to complete this survey - your input is greatly appreciated. Please feel free to forward this survey to any contacts you have with expertise in the area of inner capacities and their relation to the climate crisis.

D Overview of interviewees

Type of actor/organisation	Affiliation and/or role in organisation	Country/ work focus
United Nations	Former executive Secretary of the United Nations Framework Convention on Climate Change (UNFCCC)	International
	Senior Policy Advisor; former advisor for the United Nations Framework Convention on Climate Change (UNFCCC)	International
	Senior Policy Advisor; former staff of the United Nations Framework Convention on Climate Change (UNFCCC)	International
EU institution	European Commission, environmental policy advisor and coordinator	Ireland/ EU
	European Commission, wellbeing coordinator	Sweden/ EU
	European Commission, cross-sectoral wellbeing adviser	Denmark/ EU
	European Parliament, politician	Netherlands/ EU
	European Parliament, politician	Finland/ EU
	European policy institution, director, policy advisor	United Kingdom/ EU
National parliament & other public organisation	UK Parliament, politician, head of political party	United Kingdom
	UK Parliament, politician	United Kingdom
	UK Parliament, politician	United Kingdom
	Dutch Parliament, politician	Netherlands
	Canadian government; former President of the Treasury Board, policy-maker	Canada
	Public organisation, policy advisor and educator	United Kingdom/ International
	Estonian parliament, politician	Estonia
	Belgian government, Minister, previous member of the European Parliament	Belgium / EU
	UK Parliament, politician & international policy advisor	United Kingdom/ International
Non-profit organisations & private organisations	Policy advisor and educator	United States/ International
	Energy and Climate Intelligence Unit, director, policy advisor	United Kingdom
	Founder and director of programmes, policy advisor	Germany/ International
	Founder and executive director, policy advisor	United Kingdom/ International
	Director of Policy, policy advisor and educator	United Kingdom
	Policy-advisor and activist	United States/ International
	Policy-advisor, coach, facilitator	United Kingdom, France/ International
	Policy advisor and educator	United States/ International

Note: 24 people were interviewed directly for the project. The remaining two were not available for interview, due to time constraints. In these cases, previous interviews could be used as they were directly related to the focus of the study.

E Overview of survey respondents

Sector	Roles	Number	Total number
Public sector	Policy-/decision-maker at global level	4	37
	Policy-/decision-maker at EU level	11	
	Policy-/decision-maker at national level	5	
	Policy-/decision-maker at regional or local level (e.g. city)	8	
	Policy advisors	8	
Private sector	Policy advisors, innovators, and policy advocates	12	40
Non-governmental organisation		18	
Educational body		10	
Total number		76	76

F Illustrative citations for the identified results/ themes

Table 1: The intersection of mind and climate change. Table with illustrative citations regarding Section 3.1.

THE INTERSECTION OF MIND AND CLIMATE CHANGE
**The mind – A victim of climate change (Section 3.1.1)**
**Climate anxiety**<br>● "Obviously the state of the climate can have an impact on our inner lives when we see wildfires or floods or whatever it may be, or just news reports about how bad things are going to be in the future, that activates anxiety. It may create depression."<br>● "I think that there is a manifestation of the impacts of the situation that we're in is creating a kind of drag on the overall mental health of a whole generation. And that is, you know, that's a source of real concern."<br>● "it's too big. People don't know what to do. They feel powerless (…), you know, losing sleep."<br>● "the first interaction that comes into my mind in response to that question is seeing young people taking to the streets in order to protect the environment, to call for greater action on the climate crisis. And so many of their banners have messages like, 'I want a future, please don't destroy my future'. 'I want to live.' And it just feels the emotional toll of genuinely having a moment to reflect on what it really means to think that life as we know it on this planet is under such enormous threat. I think the mental stress associated with that is huge, and I think we're only beginning to touch the surface of it. There's a phrase that is increasingly being used around climate grief. So it's almost that there are two things going on in a way. I think one is that more people, I think, perhaps are now risking the overwhelming sense of grief and fear and powerlessness that can sometimes come from really contemplating what we are doing to the climate and how little time there is to reverse it. But for young people in particular, I really worry that going through their lives, really thinking that, for example, their own children might not have a life or some people already choosing not to have children because they think the future could be so awful. I think those kinds almost, well, they are existential and they're certainly cosmic kind of levels of anxiety is something that as a species, we've never had to deal with before, not on this scale, I mean, obviously, in times gone by there were individual threats to different countries sometimes and different groups within different countries."
**Burnout and overwhelm**<br>● "You know, I've spoken to colleagues, and I experience it myself. (…). The action that we've taken is just insufficient (…). And, you know, I think the people that work on it day to day (…) do worry about it, and carry that burden around with them (…). It's one of those things that sits in the background and gnaws away."

- “Indirect mental health effects.”
- “Depression or anxiety is just to do with the way we live and how it fails to meet our mental health needs (…), if our work doesn't give us a sense of agency or if we're faced with structural oppression and inequality.”
- “I mean, clearly, one needs a certain level of consumption (…), but beyond a certain level it leads to an undermining of wellbeing, for instance in terms of anxiety.”
- “A lot of mental health problems are because of this idea of separation, of being very isolated, very individual. Everything is my fault, my responsibility and, you know, the lack of community (…). It's a competitive system (…) and that goes with the social media and all these things where you compare yourself all the time.”
- “The anxiety is caused by our discontentment.”
- “But I but also, of course, there's the relationship with self and this massive disconnection that we are all experiencing because of the attention economy and the fact that we aren't able to relax and feel comfortable in our own skins and comfortable with silence, comfortable with being alone. But this overwhelming sense that life as we know it is under so much threat. And the species that we have the privilege of sharing this planet with are under that threat, too. I think we've only begun to scratch the surface of what that really means in terms of, how on earth do we find a sense of well-being when all of that is going on around us?”
- If our children are being depressed at 14. Seven percent of 17 year olds have tried to commit suicide in the United Kingdom. Every fourth person in the United Kingdom. Sorry, every fourth adult in the United Kingdom is either on opioids, taking sleeping tablets to sleep on anti-depressants for mental illness. Every fourth person, thirty two point three percent of fifteen to twenty five year olds have got one or more psychiatric condition, according to the question I put down in parliament. So they're beginning to realise and maybe they're realising it because it's their children, it's they themselves, it's their relatives that are feeling this. And the chicken's come home to roost. You know, when you look at all of those indicators on obesity or mental illness and even in the US and the United Kingdom now, the longevity which has increased since 1841, since the Public Health Act in 1841 has gone up. And in the United Kingdom, the United States has started to come down. And in the United Kingdom, it started to plateau because we are two of the most hyper capitalist societies in the world where the US follows the UK follows five, 10 years later and the rest of Europe will follow after that. So these are massive indicators that have been there for hundreds of years. And some on the wellbeing front and then the environmental front, you know. We're out of kilter, and it's because we are not centred.

The mind – A root cause of climate change (Section 3.1.2)

The role of the mind in causing climate change

- “It's the human mind that is at the heart of the climate crisis, even though it's often not mentioned or spoken about.”
- “One obvious but interesting insight is that nobody's trying to warm the climate (…). It is a sort of an unintended consequence of the visible manifestation of the life that our minds have created.”
- “It is the manifestation of generations of exploitative mental habits that have almost inevitably got us to this point.”

- “The climate crisis has come about because of two centuries of industrialisation without thinking about the human consequences of it.”
- “It's simply [based on] a model of economic development, which has had no regard for the impact on nature, but also really no regard for the impact on human beings. It involved slavery (…), mass factory work of an incredibly monotonous nature. It has involved treating them literally (…) just as a factor of production and ignoring what they need and (…) the value of the individual (…).”
- “I mean, we're brought up with values that are that there's enough of everything and we need the eternal growth and just go out there and, you know, whether the way we do holidays travelling (…) I think a lot of mental health problems are because of this idea of separation, of being very isolated, very individual. Everything is my fault, my responsibility and, you know, the lack of community.”
- “There isn’t anything inherent in the mind that means that it has to be that way at all. Far from it.”
- “Humans are naturally kind and concerned for others, not just other humans, but other living things and the environment. But we live in a society that rewards and celebrates generally the worst in human society about being selfish, self-interested, putting yourself first, doing what's best for you.”

Disconnection

- “We have been disconnecting ourselves for centuries now. So you could say rather successfully, but with the high price, which we start to pay more and more.”
- “The climate crisis stems from a disconnected relationship that we have as people with nature in a broad sense: with our own bodies, with the people around us, with the ecosystems we are part of, although we believe that we are living in them, you know, this distinction that's being made. It's about how we see ourselves in the world and how we see the world around us. That's to me the root cause of the climate crisis. And it can be quite obvious intuitively. But even when we work on it on the scientific side. I've been working on climate change for a long time on the mitigation side, and on the adaptation side, and if you only look at emissions or, you know, at specific extreme events that are happening today and for which we need to either, you know, find technological fixes both to reduce emissions in the transport sector, in the industrial sector, or to address, for instance, sea level rise with more dykes. If you look at those solutions, you can very quickly see their own limitations in addressing the issue in the sense that the issue is so much bigger (…). And so very quickly, you will go back to, you know, why do we think in terms of technological fixes? (…) And if you don't start with the mind and this issue of disconnection from nature, you remain in the same type of thinking that you had before.”
- “But of course, there's the relationship with self and this massive disconnection that we are all experiencing because of the attention economy and the fact that we aren't able to relax and feel comfortable in our own skins and comfortable with silence, comfortable with being alone. And COVID has, of course, exacerbated that because loneliness and lack of human connexion makes people feel even more disconnected with themselves as well as with others. (…)”
- “We humans tend to think of ourselves as the most important species on the planet. Being so self-centred is the main reason we destroy the environment. The only sustainable solution will be to let go of this mindset, this costly illusion that we are outside of nature, even the rulers of nature”.
- ”We have the climate crisis because of our inner crisis.”
- “There is this growing fear that we are being alienated from our physical reality.”

Effects of our current economic paradigm

- Our minds have become greedy and our minds have become acquisitive”
- ” Our minds are focussed on materialism, consumerism, production, economic growth. Inner discontent is reflected outside in our greed; endless pursuit of material possessions. Even if we don’t need them, we go on producing them and selling them and buying them and holding them.”
- “It’s about expectations of life, of what success looks like (…) and how you get your needs met.”
- “The commercial world that we live in does that through advertisements. The social media does that. Attention is the new gold (…). And they are forever trying to take our attention to buy this and buy that. And artificial intelligence is allowing those companies to know our minds better than we know it ourselves.”
- “That is that distraction, that lack of connection, which has allowed us to get into this state, made us worship money and to not value the things that really connect us.”
- “All of our institutions and our networks and the way we think about these issues is predicated on this rigid, huge wall between inner and outer.”

The mind – A barrier for climate action (Section 3.1.3)

Biases

- “The way we treat people in the out-group, and then making all of nature the out-group. And studies of the brain [show that] you stop having compassion for something you put in the outgroup. So whether it's a person you've deemed in a different racial group or a species that isn't your species, you treat them just like they're in the way and they're just incidental garbage. And so we treat Earth like a trash can, because of this mental state.”
- “If your horizons are in your local area [in-group], you feel utterly powerless against, you know, the magnitude of the problem and the fact that it's happening all over the world. And therefore, you know, you get a number of reactions to that. One is to blame others for it.”
- “There is no feeling that you can make that change, that you can make that difference.”
- “And when we are in a fight-flight-freeze kind of mindset, then (…) that can make us less empathetic. It can make us more prone to extremist views, more prone to pronounced in-group bias and them-and-us dynamics, all of which reduces the political space for collective action on shared problems, above all, climate change.”
- “So there's a big psychological distance between these people and those that are really being impacted the worst. And you see it in terms of, I guess, support for development aid.”
- “We humans tend to think of ourselves as the most important species on the planet. Being so self-centred is the main reason we destroy the environment. The only sustainable solution will be to let go of this mindset, this costly illusion that we are outside of nature, even the rulers of nature”.

Climate avoidance and denial

- “The climate crisis is the ultimate existential crisis. (…) It's very unusual to find somebody (…) who hasn't become conscious of their own mortality. However, it's equally unusual to find somebody who hasn't repressed, pushed away and not dealt with that (…). We repress or we grow.”
- “People don't like to be anxious, (…) but the avoidance of anxiety leads to denial (…) that leads to [increased anxiety or] other mental distress like panic disorder”
- “The real issue is that the world is in a great deterioration that we all want to try to deny and sadly in the denial then we do business as usual and we not only reinforce the problem, we make the problem soon irreversible.”
- “Yeah, well, I think people don't like to be anxious.”
- So I think people are realizing that while no one likes to be anxious, the avoidance of anxiety leads to denial and that eco-anxiety or climate anxiety, because it can’t be named, can lead to other, I think, mental distress like my friend’s panic disorder.”…….. the real issue is not the tunnel or the bridge. The real issue is that the world is in a great deterioration that we all want to try to deny and sadly in the denial then we do business as usual and we not only reinforce the problem, we make the problem soon irreversible. (…) So all of those are mental processes of avoiding exactly what you’re saying, the anxiety of realizing it (…). So what’s shocking is like even Al Gore gets the Nobel Prize and gets an Academy Award for An Inconvenient Truth. And less people are interested because they don’t want the inconvenient anxiety. And the mind, the human mind, it comes up again because it focuses on short term benefits and not long term outcomes. And so it’s that tendency of the, sadly, our human mind to do that and then to have in-group–out-group distinctions where you make your circle of compassionate concern smaller when you feel threatened, you know, then. These mental processes are not they’re not going to be easy to deal with, but they must be dealt with because they’re at the root of the perpetuation of the problem, if not the creation of the problem.”
- “One of the reasons that we’re in this crisis is that people don’t even have the time, or perhaps the courage, to feel what we’re doing to nature and the climate and our planetary home. Unless one can make that connection then, in a sense, it’s hard to find the resourcefulness to try to change things, and you’re part of the problem.”

Short-term thinking, cognitive versus embodied thinking

- “The COVID-19 pandemic is a showcase of the climate crisis in small letters. It shows that the human mind is so poor in dealing with long-term issues and delays in impacts.”
- “We act very much based on our feelings, and what we feel is more based on experience than knowledge.”
- “I think what’s needed is to understand the human being as a holistic thing... What is my body, my mind or my feelings? They cannot exist without this whole environment that I subsist in. There is no fish without water. There’s no human without this planet. I am of this planet.”

The vicious cycle of mind and climate change (Section 3.1.4)

Feedback loops

- “It’s kind of a vicious cycle type of logic. The fact that we’ve lost this connection to a more holistic way of looking at the world creates a crisis that will get back to us through anxiety or stress, or through direct physical impacts and our health.”
- “There are feedback loops between the state of the world and our states of mind, which we need to get better at both recognising and acting on. So, I mean, the climate is in a way the best and the easiest place we can illustrate that. So obviously the state of the climate can have an impact on our inner lives when we see wildfires or floods or whatever it may be, or just news reports about how bad things are going to be in the future, that activates anxiety. It may create depression. There’s all the stuff that we know about climate grief and so on. (…) But then there is the other side of that feedback loop. So in a sustainability context, the obvious example would be that, you know, very often when we feel depressed or anxious or lonely or bored, all state of mind things, we may go shopping to make us feel better. It’s kind of almost like the most universal modern ritual. And obviously, that has direct implications for unsustainable consumption. But it also affects how we behave as citizens in democracies. And when we are in a fight-flight-freeze kind of mindset, which, again, climate impacts can induce, that can make us less empathetic. It can make us more prone to extremist views, more prone to pronounced in-group bias and them-and-us dynamics, all of which reduces the political space for collective action on shared problems, above all, climate change.”
- “But those two poles are creating a tension. There’s the presence of it, the inability to look at it. The anxiety that is engendered as a result of that creates a little loop that becomes self-reinforcing and gets worse.”
- “there is a growing sense that we’re on the wrong track, that we’re following, by being so fixated, for example, on GDP growth as the be all and end all of our economic life, it’s not even working in its own terms. And at the same time, it’s utterly destroying the planet. And so it feels that the movement to give more time and space and privilege to those sort of inner human qualities actually is a matter of life and death, it’s a matter of life and death for us individually in order to be able to try to navigate our way through this incredibly difficult time. But also, if those sorts of values were prioritised more, then it might just get us off this treadmill that is literally destroying life on our planet.”
- “So there’s an interesting, like, vicious cycle that can become a virtuous cycle”
- “Consumption we know that after a certain level doesn’t actually increase people’s well-being at all. I mean, clearly, one needs a certain level of consumption in order to be able to meet your needs and to have a flourishing life, but beyond a certain level, the idea that more and more growth, more and more chasing of material goods is going to increase your well-being, I think has been shown so many times not to be the case. The New Economics Foundation, for example, a think tank here in the UK, has done quite a lot of work showing that beyond a certain level of consumption, actually, more and more growth after that point leads to greater, not only does it not lead to wellbeing, but it leads to an undermining of wellbeing, in terms of anxiety, in terms of air pollution, in terms of, you know, streets being too busy to be able to walk down and just not having the hope in the future for your kids.”
- “When I was working on Brexit and I became really interested in Cambridge Analytica because obviously what Cambridge Analytica was able to do was use a mixture of psychological profiling and then social media microtargeting, in effect, to weaponize our own anxieties against us. So with a very, very high degree of accuracy, CA would be able to use data about you individually and then get messages into your Facebook feed to trigger you to see the world in them-and-us terms just when it counted during an election campaign.”

Table 2: Links to current climate policy. Table with illustrative citations regarding Section 3.2.

LINKS TO CURRENT CLIMATE POLICY APPROACHES
**Lack of integrated approaches – fuelling the vicious cycle of mind and climate change (Section 3.2.1)**
**Inner capacities not considered in policymaking**<br>● "There is a divorce between inner and outer."<br>● "We've tried to connect to the climate crisis from the head. We've tried to connect with science, with statistics, with analysis, with threats, with scares (…), without success."<br>● "You see that struggle in [the IPCC] working group three where they try to go to the next step: Why is nothing happening? Why are we every time running into opposition? Why is making the changes happen so difficult? (…) IPCC is trying to get more into that. And you see it also with the European Commission, who is trying in the Green Deal to explicitly talk about actors of change, and in this entire idea of the Climate Pact that they launched (…). However, it is often only talking."<br>● "The IPCC conclusions haven't changed in 15 years. 'There is climate change. Humans are causing it. It has great impacts. We have policy instruments to solve it at a reasonable cost.' Why is it not happening? Why is making changes so difficult? The IPCC and European Commision are now trying more to understand that... how can these elements of inner qualities and capacities be included?"<br>● "There's a need for looking into how can capacities and inner qualities be used"<br>● UNESCO they talk a lot about mindsets and it's all about how war started in the mind of man. And so like the war on nature also started in the mind. So the discourse is pretty much about mindsets. But then I don't see. And they're also working on indigenous knowledge systems that can challenge the Western modern mindset, but they don't work specifically with mindfulness, for instance. And I see it more as discourse at the moment than as practices<br>● "Climate change action in the past has been disrupted or jettisoned by financial crises, which indicates that for some of the population including key decision-makers in government and business and finance, the financial system and the economy has at times trumped the climate change action (…) But today, the traction on fighting climate change is actually being driven by people who come from, leaders from the business and financial sector. So I actually think these market based capitalistic thinkers have actually, in some ways by combining the self-interest that is inherently part of the market, and by aligning those self-interests with tackling climate change, we might actually have a formula that is that that actually works"

- “We need to approach the climate crisis from a position of consciousness, and of conscience, as well as science.”

Insufficiency of current approaches

- “It’s about trying to understand, essentially, how you nudge people towards being able to do things that contribute to emissions reduction.”
- “We don't get much more beyond these very small nudges.”
- “And you see people jumping on it, oh, yeah, let's discuss nudging now for the next 20 years,… come on… This should not be another delaying tactic!”
- “People are only just starting to understand [the broader linkages between mind and climate change]”
- “Environmental issues are a bonus that you can have when everything else is going well, but as soon as things get a little bit difficult or there's another incentive to do something else, then you know that that was a luxury we can now put aside.”
- “It focuses on the outer – technology, industrialisation, economic growth (…). They want to fix the problem with more technology, more renewable energy, more solar power, more nuclear power, more wind power (…). But you cannot solve the problem with the same minds or mindsets that created the problem in the first place. (…) Science and technology have been the instruments of producing industrialisation, industrial revolution and materialism and ever increasing economic growth. And now we want to solve that problem by another kind of technology.”
- “Politics at the moment, with the kind of return to nationalism and to us-versus-them politics, has actually reduced the connection between people in the sense of it's reduced the community. The community has shrunk back to the people like me, the people I can either empathise with because they're exactly like me and never mind the others. They're the enemy.”
- It’s about “me, myself, my mandate and my term of political influence”
- “Emotion is stripped out of it (…). You immediately get into a discussion on instruments (…), instead of the intrinsic motivation on why are we doing that?”
- “You've had 50 years of amazing innovation in activism, the kind of birth of modern feminism, environmentalism, so much other stuff besides but often without the necessary inner work. And so you get what we have now, what we see all around us in the non-profit sector, that you have chronic burnout.”
- [Inner-outer work are] “more a discourse at the moment than a practice.”
- “The consideration of the intersection between mind and climate in policymaking is twofold. It involves the policy that is made and in what impact they might have (on the wider world and related inner qualities), and the process of policymaking and the qualities that are needed to make that policy in the first place. Other wording: Related to policymaking (content and impact of policies & the process of policy-making and the people and their inner qualities/ capacities involved in this.”
- “As long as the process was continuing, as long as you were finding technical solutions to problems, … financial mechanisms… But the whole human dimension everywhere was missing. (…) This kind of like ‘means to an end logic’ didn't make any sense.”

Isolated advances that challenge current approaches (Section 3.2.2)

Recent policy interest in inner aspects/ wellbeing

- "If people have mental resources, if they have wellbeing, then they have also so much more resources to work against the climate crisis, and empathy towards other people (…) less aggression, less racism, less stereotypes, and more resources to work in a positive way towards the planet.. This is why I find it important to push for s mental health initiatives, internally within the organisation and also in external policy work."
- "There are things like the way that the British government over the last 10 years has become much more interested in wellbeing, for example (…). But that's not climate-specific."
- "There's still very much a focus on the individual wellbeing, as if it's almost like a medical problem. (…) There's so much more to that. But it is a good way in, and it's a way of getting people to start [inner and outer spheres of transformation]."
- "My current project actually at the moment (…) I say, if you're still alive in 20 years, this is the way it's going to be, and this was before COVID, I said most of you will spend most of your time with your parents helping them, working, working the crops and growing the vegetables, you'll hang around with your mates in the park, having a bit of a laugh. And if you're very unlucky, some of you will learn to play the guitar and sing folk songs. There will be one screen in your house. It'll be on for about two hours a day. There will be no, everyone will shit in compostable toilets because we would be completely unable to keep anything like a sewage system going and so on and so forth. You will never step on board an aeroplane and you will walk to and from to everything or you will be on a bicycle. And I said that would give you a point zero zero zero one per cent of still being on this planet in 20 years. The amazing thing is when I give that vision to kids, they say, fuck, sounds okay to me. But the box who are the adults who are running the system, this is incomprehensible to them. (…) So I mean what I'm trying to do at the moment (…) So literally trying to come up with a situation where I can sort of say, OK. It's pretty much the same thing they did in Ireland for gay marriage. Like, you know, you break it into kind of manageable groups, either in counties or communities. And you have a tactic of sort of saying, OK, look, we have to get here, you know, so so in climate change, in climate terms, we have to do unimaginable cuts in our emissions while at the same time already drawing down massive amounts of CO2. It's almost impossible to imagine how we'd do this. So the change is huge. So what could we agree on? So let's say, it's not quite this, but let's say it's one-third of your carbon emissions are what you eat. One-third of your carbon emissions are keeping a house going and one-third of your carbon emissions are your work and transport. OK, how do we identify, which bits would you be prepared to lose? (…) to design the questionnaire for kids. What would you lose? OK, Mommy and Daddy won't be able to drive me to work, to school anymore. OK, meat once a week. It'll actually be rationed. OK, so literally have a list of things. Say this is what, 12 to 19-year-olds say they will be prepared to do. (…) "The most powerless people on this earth are the ones you find at the very top of organisations because they got there by being totally part of the system. So I'm really interested in seeing if we couldn't get the kids to help design the questionnaire as to what the kids would be prepared to put up with and how and how quickly. (…) So like, is that maybe a way in, to actually give a voice to these next generations? How could that work? Because the politicians can't lead, but they can do the managing when society decides where to go."
- [Advances using IT:] "Start-Up, where people who own land or forest can basically get these kind of like tokens to not interfere with this environment, to not sell their forest off, but instead, they get money from companies who want to better their ecological footprint, so to say, but I see it as also the Start-Up community has a will to do something for the environment. So maybe this kind of link between the digital and the physical worlds where there is a lot of also potential obviously to actually

work with this heightened awareness in the developed countries, especially, of course. But I think also in all of the world, the use of digital devices is growing so rapidly."

Individual pioneers of change: Between political marginalisation and concealed endurance (Section 3.2.3)

The difficulty of effecting change within current structures/ paradigms

- "I've seen individual bright spots of particular people who are able to cut through, but I haven't seen institutional structures that create that [integration] to be honest."
- "As long as the process was continuing, as long as you were finding technical solutions to problems, … financial mechanisms… But the whole human dimension everywhere was missing. (…) This kind of like 'means to an end logic' didn't make any sense."
- "I decided to leave to do something meaningful."
- "I know my colleague always tries to put in things. And it's always taken out, you know. … (…) But he's kind of, 'yeah, but eventually maybe somebody will miss it and it goes through'. He's insisting, insisting."
- "Up it gets blocked."
- "Trying to change the top (…) is not going to happened, (…) so then we will have to go from down to change upwards."
- "I'm at the realization that if we could get quantity on the grassroots level, (…) maybe then the commissioners will actually listen."
- "Like, my boss will come to me and sort of say, I see you're holding yet another climate conference (…) with kids from European schools. Yep, you want to stop me? No, it's too late, but it's not part of my job. So it's never in the direct line of my job. The direct line of my job would be please invite around the chip manufacturers and the large platforms to see how we can sensibilise people to the effects that their searching habits online might... Complete and utter waste of time, you know."
- "I've been told I will be fired if I share it" (information related to making the inner a more predominant part of climate action, and giving more agency to individuals)"

Creation of support groups/ the field

- "I think the role of community and human relationships matter so much more, both in terms of the building of the resilience that we need in order to cope with the crisis. The sense of being on your own and doing that, I think is pretty overwhelming, whereas reaching out in different kinds of support groups or, you know, whatever kind of grouping of like-minded individuals you have around you can give both enormous strength to allow one both to feel the grief, but then to kind of try to find the resources to respond to it and to try to mobilise and campaign against it and so forth. So I think partly it is that real recognition that we're not alone in this, and the more that we can reach out to one another, then the strength one gets from that I think is huge."

Engaging in practices to aid work

- “It's changed my focus on my work to be about the climate crisis and about social justice. I see those as fundamentally the same thing, really, you know, separation leading to these destructive processes.”
- “It was important to go into this inner dimension of the crisis. (…) It was some kind of no return point, like I couldn't go back to doing the work the way it was done before, not to consider that [inner dimensions] or to see it as an aside from the important work. (…) And then everything changed. Everything changed in how I interacted with institutions, people and what I wanted to work on.”
- “I just felt that I have more to give and I can give it maybe on a bigger scale.”
- “Being in the movement from possibility to probability to actuality.”
- “I couldn't stand it anymore because it was just too overwhelming, too negative, and I just started working with myself.”

Table 3: Future visions. Table with illustrative citations regarding Section 3.3.

FUTURE VISIONS
**Contextual barriers and drivers (Section 3.3.1)**
**Social paradigms underlying climate change**<br>• “The solution to the challenge is to draw that out of the subconscious and make it visible, which will then revert back to changing our behaviour in relation to it. And that's proved very hard to do. People have proved very resistant to that dragging out and making that visible. So how that happens in a manner that doesn't make people feel threatened or challenged, but enables them to feel empowered as part of that process is really important.”<br>• “Requires enormous leadership to show people that they could be happy with a different kind of lifestyle and that the current model is not meeting their real needs, including emotional needs, social needs and fulfilment.”<br>• “All these organizations suffer from power games played at the top and powerlessness at lower levels, from infighting and bureaucracy, from endless meetings and a seemingly never-ending succession of change and cost-cutting programs.”<br>• “The most powerless people on this earth are the ones you find at the very top of organisations because they got there by being totally part of the system.”<br>• “The policy is developed by powerful political lobbies. (…) The pharmaceutical industry is worth a half a trillion dollars a year. Five hundred billion dollars a year. Well, these statistics come from a few years back now, and they spend 20 billion pounds a year on government relations and public relations and trying to influence

decision-makers. And that's just the pharmaceutical industry. When you look at the oil and gas industry, you've got billions, if not hundreds of billions of pounds spent trying to make decision-makers bend this way or bend that way."

Media and digital technology

- "The digital communities have a positive role to play in this maybe. (…) I think when industrialisation took away from people the need for much physical labour, then a kind of a culture for physical exercise appeared. (…) If we see now that the digital devices have changed the means of how our senses are stimulated, or how we use our senses, then there should also be a counterforce, maybe a kind of mental training of the mind. How are we able to cope with the amount of pressure that is put on our attention, digital devices and this kind of stimuli? There is a parallel to what happened during industrialisation and physical labour. Now we have to deal more with the attention and the capabilities of the mind."
- "A way in, to actually give a [stronger] voice to these next generations. How could that work? Because the politicians can't lead, but they can do the managing when society decides where to go."
- "We are so focussed on digital stimuli that as a counterforce we have a growing need to look inside, and also to look to real and physical things such as nature. There is this growing fear that we are being alienated from our physical reality."

Covid and other societal crises

- "The current pandemic has made it [climate change] an even bigger issue because people have become more aware of the interconnectivity and the global nature of challenges in our everyday lives."

The mind – gateway/ level for change / support of transformative qualities as a gateway for individual and planetary flourishing and transformation (Section 3.3.2)

The mind as a gateway for change

- "The one approach of the many, many approaches that are needed is to work at the level of the mind."
- "These mental processes are not going to be easy to deal with, but they must be dealt with because they're at the root of the perpetuation, if not the creation of the problem."
- "You have to realise at quite a fundamental level that you're constructing your world through (…) the filter that you put everything through. So softening that requires you to have a tiny bit of space between phenomena and mental reaction, and that requires quite a bit of mental discipline to do that and to realise that (…) as you meet the world."
- "How do you transcend a paradigm that you are operating inside?"

- “It feels that the movement to give more time and space and privilege to those sort of inner human qualities actually is a matter of life and death, it's a matter of life and death for us individually in order to be able to try to navigate our way through this incredibly difficult time. But also, if those sorts of values were prioritised more, then it might just get us off this treadmill that is literally destroying life on our planet.”
- “An attempt to break a mental habit of ignoring something and then feeling anxious about it and then ignoring it more and feeling anxious about it. (…) Instead of that sort of rejection and aversion, you make it something that feels like a great shared endeavour that is inspiring.”
- “It can flip people's habits from a sense of powerlessness, not being able to participate, it's all too big… to quite a different sense of participation and meaning. I think in that there's one particular dynamic that we've gotten wrong quite significantly, and that's the relationship between meaning and control. So in most areas of human life, meaning has nothing to do with control and actually meaning can come from an absence of control. (…) In climate change, we say unless you have control, there's no meaning. You know, why are you bothering to change that? Why are you bothering to try to do X, Y and Z? What about China? (…) Actually, that was a deliberate strategy by oil and gas companies to undermine the collective response to climate change. And we need to move beyond that and start realising, actually taking action to protect the planet, to do what we can to protect future generations, to avoid ecosystem collapse is some of the most meaningful work that we can do at this moment, at whatever level is appropriate for us, even though, perhaps especially because, we can't control the outcome.”
- “How we deal with the global challenges in this decisive decade will be determined by who we are and how we're able to show up to that moment.”
- “My experience of international diplomacy, which is of the UN and negotiations between countries, is that treaties are negotiated by people, not by countries, and progress forward is determined far more by individual characteristics, individual styles, individual relationships than is evident at all.”
- “An important finding from existing advances is that when it comes to intrinsic values, it doesn't matter which one you are working with, whether it's working for the environment, working for equality, (…), the values are all linked together. So if you start activating one, it will activate all the others. (…) All of them pull your society in the direction of caring.”
- “There are many changes to make over the next 10 years, and each of us will take different steps along the way, but all of us start the transformation in one place: our mindset.”
- “We’re swamped with so much information, so cultivating attention is key - the ability to focus on and retain information so as to really discern what is important.”
- “The first ability that we should cultivate to support climate action is the ability to connect… to our own bodies, to others, to nature around us… But what emotions then come can be unpredictable, so the second ability would be to feel them without shutting down or recreating the distance”
- We need to approach the climate crisis from a position of consciousness, and of conscience, as well as science. I think that’s where mindfulness and compassion can be absolutely key. They can make that connection between the mind, body and heart - in an evidence-based way.”

From vicious to virtuous cycles of mind and climate change (Section 3.3.3)

Integrating considerations of mind in climate policy and action

- “There's an interesting vicious cycle [of mind and climate change] that can become a virtuous cycle.”
- “Reverse the polarity of that [vicious] cycle.”
- “Progress on the state of the world, whether that's addressing climate or reducing poverty or whatever it may be, [in a way] that makes us feel more open, secure, hopeful, which leads to more prosocial forms of engagement as citizens, and that increases the capacity for collective action.
- “Mainstreaming approaches can be of guidance.”
- “Obviously, we were all over issues like gender equity and climate and many other things that needed to be mainstreamed. (…) You are embedding traditions and rituals and practises that will sort of become more and more embedded as the bedrock of something bigger.”
- “It's taken a long time with gender equality to create the structures. (…) But something like this, how you do this, I think this becomes a manifestation of the underlying culture. I think that it's the underlying culture that needs to evolve. And then the institution-building follows culture.”
- “Building a field was really crucial for things like reducing teen smoking or reducing malaria, because what you're doing is creating both a sense of group identity, but also the networks for kind of intensive learning and knowledge sharing, and then you're creating an ecosystem that funders can begin to support. I think something like that is needed here because obviously, the kind of practitioners that are relevant are going to be educators and health workers and social workers and central government and employers. And, you know, the list is so long. And I think that, you know, starting with some pilot projects would be a good place to start, which I'm imagining might be place-based maybe at the local level”
- “And our role there is often to talk about the psychological aspects of campaigning, particularly to put to these organisations the argument that, a lot of their communications work deliberately triggers people because, you know, that's good for firing up your supporters or getting media hits or getting funding. But our argument is that that comes at a significant price, that every time we trigger our members with very upsetting imagery of kind of starving children or whatever it may be, we are, in effect, taking people into a fight-flight-freeze reaction that then deepens political polarisation. And we cannot solve climate change if we're hyperpolarized. (…) (…) they're constantly winning battles and losing the war.
- “If we're going to make the changes of the sizes that have to be made, they must be made in some sort of coherent and agreed way. OK, and so how do you do that?” (linked to creating access to data that allows to include individuals, give individuals more agency and voice).”
- “(…) people who own land or forest can basically get these kind of like tokens to not interfere with this environment, to not sell their forest off, but instead, they get money from companies who want to better their ecological footprint, so to say, but I see it as also the Start-Up community has a will to do something for the environment. So maybe this kind of link between the digital and the physical worlds where there is a lot of also potential obviously to actually work with this heightened awareness in the developed countries, especially, of course. But I think also in all of the world, the use of digital devices is growing so rapidly.”
- “The solution to the challenge is to draw [our social paradigm] out of the subconscious and make it visible, which will then revert back to changing our behaviour in relation to it. And that’s proved very hard to do. People have proved very resistant to dragging [that] out and making [it] visible. So how that happens in a manner that doesn’t make people feel threatened or challenged, but enables them to feel empowered as part of that process is really important”. This “requires enormous leadership

to show people that they could be happy with a different kind of lifestyle, and that the current model is not meeting their real needs, including emotional needs, social needs and fulfilment".

Integration of sustainability and wellbeing issues in education policy

- "Being able to teach about sustainable development and not just the knowledge of it, but the values and attitudes that went around it."
- "Learning for sustainability is made an entitlement for every school child. They're also entitled to outdoor learning as a regular and progressive part of their education."
- "The teacher training standards"
- "Those public policy areas have been health, education, criminal justice and the workplace. There's a massive missing strand there, which is the environment and climate."

G Additional figures regarding the described mind-climate change nexus

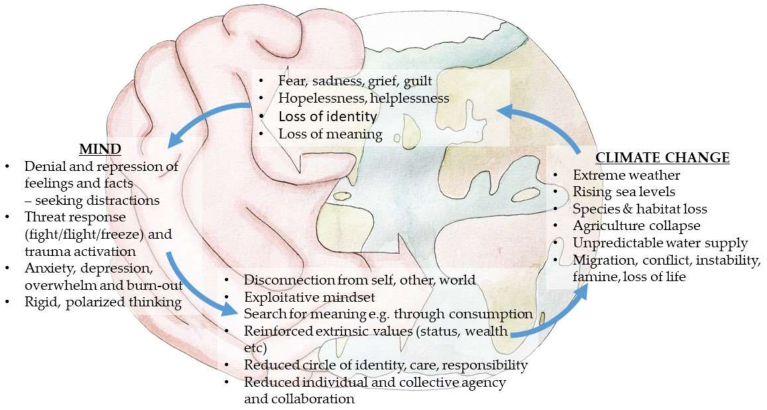

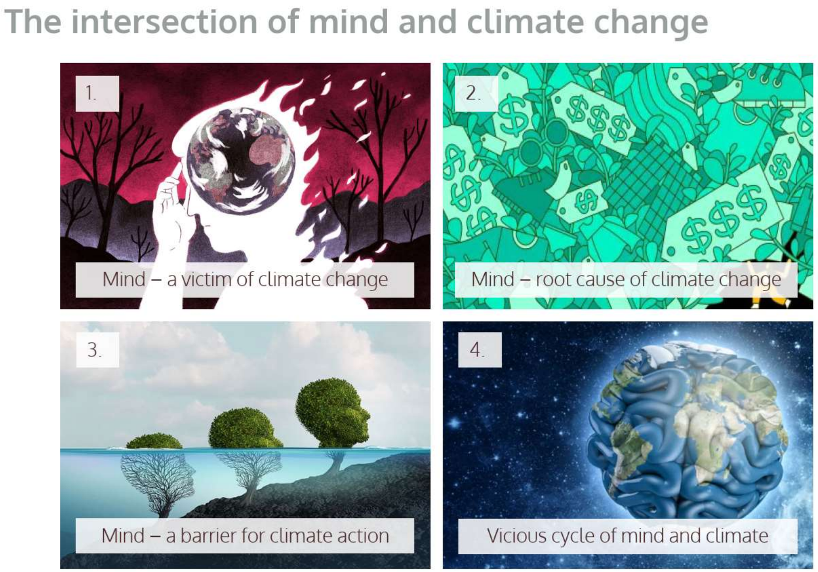

H Analyses of transformative qualities/capacities

Exploratory analysis of mentioned transformative qualities/capacities (TC); categories based on Wamsler et al. 2021

Clusters	Specific quality/capacity	Interview data on qualities/capacities (Q2)	Interview data on link to mindfulness (Q3)	Survey data on qualities/capacities (Q4 and Q5)	Survey data on link to mindfulness (Q6)
TC1: AWARENESS<br><br>Definition: The ability to meet situations, people, others and one's own thoughts and feelings with openness, presence and acceptance.	Psychological resilience	4	3	13	5
	Emotional intelligence	4	3	5	4
	Openness	5	7	15	5
	Equanimity	1	2	11	-
	Discernment	1	-	-	-
	Attention	2	3	12	4
	Acceptance	4	5	4	5
	Self-reflection	6	1	10	3
	Self-awareness	9	10	23	16
	Adaptive/ flexible response capacity	2	1	8	3
	Regulation and processing	1	4	6	8
	Presence	8	6	22	16
	Meta-cognition	1	-	2	5
	Cognitive flexibility	-	1	5	3
	Deep listening	-	1	-	-
	Total	48	47	137	78
TC2: CONNECTION<br><br>Definition: The ability and desire to see and meet oneself, others and the world with care, humility and integrity, from a place of empathy and compassion	Compassion	9	12	63	21
	Empathy	3	-	15	5
	Human-nature connectedness	9	3	13	1
	Gratitude	3	-	2	-
	Care	5	2	5	-
	Humility	5	-	3	-
	Solidarity	1	-	2	-
	Connectedness	7	7	1	4
	Integrity	1	-		-
	Seeing shared humanity	1	1	-	-
	Love	1	-	-	-

	Care	-	1	1	-
	Solidarity	-	1	-	-
	Kindness	-	2	2	-
	Generosity	-	-	1	-
	Total	45	29	52	20
TC3: INSIGHT<br><br>Definition: The ability to see, understand and bring in more perspectives for a broader, relational understanding of oneself, others and the whole	Valuing diversity	1	-	-	-
	Relational awareness/ thinking	2	1	1	-
	Perspective taking	3	6	4	-
	Sensemaking	1	3	4	-
	Perspective-seeking	1	4	-	-
	Care and forgiveness regarding our history	1	-	-	-
	Integral thinking	2	-	4	-
	Integrating different ways of knowing	1	3	-	-
	Sense of reciprocity, inter/ intra-connectedness	Linked to above qualities	-	-	-
	Total	12	17	13	0
TC4: PURPOSE<br><br>Definition: The ability to navigate oneself through the world, based on insights into what is important (intrinsic, universal values)	Meaning making	1	-	-	-
	Intrinsic value orientation	3	5	4	1
	Sense of responsibility	1	1	1	-
	Future orientation	2	3	2	-
	Sense of purpose	-	1	-	-
	Equitable thinking	-	1	-	-
	Total	7	11	7	1
TC5: AGENCY<br><br>Definition: The ability to see and understand broader and deeper patterns and our own role in the world in this regard, and to have the intention, optimism and courage to act on it	Courage	3	3	5	-
	Creativity	3	1	-	-
	Sense of agency	3	3	4	5
	Sense of empowerment	2	3	-	1
	Hope	3	-	2	-
	Action-oriented mindset	3	3	-	4
	Solutions-based mindset	2	1	-	-
	Cooperation and co-creation	1	1	-	-
	Optimism	3	1	-	-

	Perseverance	-	2	4	1
	Total	23	18	15	11

Acknowledgements: We would like to thank Gustav Osberg and Heidi Hendersson for their support in developing this table.

Exploratory analysis of mentioned transformative qualities/capacities (or so-called inner development goals IDGs); categories based on Jordan 2021

Cluster	Specific quality/capacity	Interview data on qualities/capacities (Q2)	Interview data on link to mindfulness (Q3)	Survey data on qualities/capacities (Q4 and Q5)	Survey 1 data on link to mindfulness (Q6)
IDG1: BEING	Self-awareness	9	10	27	15
	Openness and learning mindset	6	7	26	7
	Presence	8	6	27	15
	Inner compass	4	5	4	-
	Integrity and authenticity	-	-	6	1
	Total	27	28	90	38
IDG2: THINKING	Long-term orientation and visioning	2	3	4	-
	Critical thinking	4	1	2	-
	Sense-making	1	3	-	-
	Perspective skills	3	6	5	-
	Complexity awareness	-	2	13	1
	Total	10	15	24	1
IDG3: RELATING	Humility	5	-	7	1
	Appreciation	2	-	2	-
	Empathy and compassion	7	12	76	21
	Connectedness	14	7	32	6
	Total	28	19	113	28
IDG4: COLLABORATING	Communication skills	3	3	1	-
	Co-creation skills	1	2	5	-
	Inclusive mindset and inter-cultural competence	2	-	5	2
	Trust	-	1	5	2
	Mobilisation skills	-	-	-	2
	Total	6	6	16	6

IDG5: ACTING	Courage	3	4	15	1
	Creativity	3	2	3	1
	Optimism	3	1	3	-
	Perseverance	1	2	6	4
	Total	10	8	15	6

Acknowledgements: We would like to thank Gustav Osberg and Heidi Hendersson for their support in developing this table.

I Climate Policy Mainstreaming Framework

Analysis framework for systematising mentioned integrative measures and approaches, categories based on Runhaar et al. 2018 and Wamsler 2015*

Complementary mainstreaming strategies and measures	Aim of mainstreaming strategies and measures
Strategy I – Add-on mainstreaming:	Establishment of specific on-the-ground projects or measures to address climate change at the local level that are *not* an integral part of the organisation's/ department's core objectives, but directly target climate change or related aspects.
Strategy II – Programmatic mainstreaming:	Integration of aspects of climate change into the organisation's/ department's core work, i.e. into their local on-the-ground projects or measures. The aim is to improve/ modify projects/ measures in order to reduce their likelihood of increasing climate change and risk, and to maximise their potential to reduce risk, mitigate and adapt.
Strategy III – Organisational (managerial, regulatory and intra-organisational) mainstreaming	Modification of the organisation's management, policy, corpus of legislation, working structures, internal education, and tools that ensure the integration and institutionalisation of climate change adaptation and mitigation at the local level.
Strategy IV – Internal mainstreaming	Modification of the organisation's way of operating and its internal policies to reduce its risk and ensure its continuous functioning in a context of increasing climate change and associated impacts.
Strategy V – Inter-organisational mainstreaming for risk governance	Promotion of collaboration between the organisation and other stakeholders (international, regional and local governmental, and civil society) to generate shared knowledge, develop competence and take joint actions to advance climate change adaptation and mitigation, and their integration.
Strategy VI – Educational mainstreaming	Support for a conceptual shift (individual and collective/ cultural) in the philosophy that drives relevant education and stewardship. The aim is that climate change considerations become inherent to all sectors and spheres of activities.

* Mainstreaming is proposed when seeking a coherent approach to address climate change. The recent IPCC report (2022a) notes with *high confidence* that sustained actions are strengthened by mainstreaming climate considerations into, for instance, institutional budget and policy planning cycles, statutory planning, and monitoring and evaluation frameworks, along with local efforts. In the same report, the term mainstreaming is mentioned 228 times. Please also see Table 1 in the article for illustrative examples of the listed strategies.
